# Oxysterols Protect Epithelial Cells Against Pore-Forming Toxins

**DOI:** 10.3389/fimmu.2022.815775

**Published:** 2022-01-26

**Authors:** Thomas J. R. Ormsby, Sian E. Owens, Liam Clement, Tom J. Mills, James G. Cronin, John J. Bromfield, Iain Martin Sheldon

**Affiliations:** ^1^ Swansea University Medical School, Swansea University, Swansea, United Kingdom; ^2^ Department of Animal Sciences, University of Florida, Gainesville, FL, United States

**Keywords:** oxysterol, epithelial cells, cholesterol, liver X receptor, pore-forming toxins, cholesterol-dependent cytolysin, cytoprotection

## Abstract

Many species of bacteria produce toxins such as cholesterol-dependent cytolysins that form pores in cell membranes. Membrane pores facilitate infection by releasing nutrients, delivering virulence factors, and causing lytic cell damage - cytolysis. Oxysterols are oxidized forms of cholesterol that regulate cellular cholesterol and alter immune responses to bacteria. Whether oxysterols also influence the protection of cells against pore-forming toxins is unresolved. Here we tested the hypothesis that oxysterols stimulate the intrinsic protection of epithelial cells against damage caused by cholesterol-dependent cytolysins. We treated epithelial cells with oxysterols and then challenged them with the cholesterol-dependent cytolysin, pyolysin. Treating HeLa cells with 27-hydroxycholesterol, 25-hydroxycholesterol, 7α-hydroxycholesterol, or 7β-hydroxycholesterol reduced pyolysin-induced leakage of lactate dehydrogenase and reduced pyolysin-induced cytolysis. Specifically, treatment with 10 ng/ml 27-hydroxycholesterol for 24 h reduced pyolysin-induced lactate dehydrogenase leakage by 88%, and reduced cytolysis from 74% to 1%. Treating HeLa cells with 27-hydroxycholesterol also reduced pyolysin-induced leakage of potassium ions, prevented mitogen-activated protein kinase cell stress responses, and limited alterations in the cytoskeleton. Furthermore, 27-hydroxycholesterol reduced pyolysin-induced damage in lung and liver epithelial cells, and protected against the cytolysins streptolysin O and *Staphylococcus aureus* α-hemolysin. Although oxysterols regulate cellular cholesterol by activating liver X receptors, cytoprotection did not depend on liver X receptors or changes in total cellular cholesterol. However, oxysterol cytoprotection was partially dependent on acyl-CoA:cholesterol acyltransferase (ACAT) reducing accessible cholesterol in cell membranes. Collectively, these findings imply that oxysterols stimulate the intrinsic protection of epithelial cells against pore-forming toxins and may help protect tissues against pathogenic bacteria.

## 1 Introduction

Many species of pathogenic bacteria produce toxins that form pores in the cell membrane of eukaryotic cells (1, 2). These pores facilitate infection by releasing nutrients, delivering virulence factors, and causing lytic cell damage - cytolysis. The most common pore-forming toxins are cholesterol-dependent cytolysins, which bind to cholesterol in cell membranes (2–5). Oxysterols are oxidized forms of cholesterol that help regulate cellular cholesterol (6, 7). In addition, some oxysterols regulate immune responses to bacteria and influence disease pathogenesis (8–10). However, an unresolved question is whether oxysterols can also alter the intrinsic protection of epithelial cells against cholesterol-dependent cytolysins.

Epithelial cells protect underlying stromal tissue cells against bacterial invasion (1, 11). However, many species of pathogenic bacteria use pore-forming toxins to damage the epithelial barrier (1–3). Within minutes, membrane pores allow potassium efflux and calcium influx, activate mitogen-activated protein kinase (MAPK) cell stress responses, and trigger membrane repair mechanisms (12–16). Within hours, membrane pores result in leakage of cytosolic proteins such as lactate dehydrogenase (LDH), cause cytoskeletal disruption, reduce cell viability and cause cytolysis (14, 17, 18). For example, *Trueperella pyogenes* secretes the cholesterol-dependent cytolysin pyolysin, which forms pores in many types of cells, causing lung, liver, skin and female reproductive tract disease in ruminants, swine and other animals (18–21). Pyolysin is secreted as 55 kDa monomers and 30 to 50 molecules oligomerize to form 18 nm internal-diameter β−barrel transmembrane pores that cause cytolysis (19, 22). However, binding of cholesterol-dependent cytolysins to cells and pore formation is sensitive to changes in cell membrane cholesterol (3, 21, 23, 24).

The majority of cell membrane cholesterol is sequestered by proteins and lipids such as sphingomyelin (5, 25). However, membrane cholesterol becomes accessible to cholesterol-dependent cytolysins when cholesterol exceeds 35 mol% of membrane lipids (4, 5). Consequently, depleting this accessible cholesterol can limit pore formation and cytolysis (18, 23). Oxysterols help maintain membrane cholesterol homeostasis by activating liver X receptors (LXRs) to increase cholesterol efflux, accelerating HMGCR degradation to reduce cholesterol biosynthesis, and stimulating acyl-CoA:cholesterol acyltransferase (ACAT) to promote cholesterol esterification (6, 7, 26). Furthermore, side-chain hydroxycholesterols such as 27-hydroxycholesterol and 25-hydroxycholesterol, and ring-modified hydroxycholesterols such as 7α-hydroxycholesterol and 7β-hydroxycholesterol, can modulate immune cell function and limit the severity of inflammation and disease (7–9, 27). Recent studies also found that 25-hydroxycholesterol stimulation of ACAT helped protect murine macrophages and bovine endometrial cells against cholesterol-dependent cytolysins (28, 29). However, understanding of oxysterol-stimulated protection of cells against cytolysins is incomplete because the ability of 25-hydroxycholesterol to limit *Listeria monocytogenes* dissemination across epithelia was not thought to involve the pathogen’s cholesterol-dependent cytolysin (10).

Here we tested the hypothesis that oxysterols stimulate the intrinsic protection of epithelial cells against damage caused by cholesterol-dependent cytolysins. We initially treated HeLa cells with oxysterols and then subsequently challenged the cells with pyolysin. Protection of cells against damage caused by pyolysin was assessed by measuring the leakage of potassium and LDH, and by evaluating MAPK stress responses, cell morphology and cell viability. Evidence for oxysterol cytoprotection was also examined for other types of epithelial cells and pore-forming toxins. In addition, we investigated whether the increased protection of cells against pyolysin following treatment with oxysterols was mechanistically associated with ion flux, LXR expression, or ACAT activity.

## 2 Materials And Methods

### 2.1 Cell Culture

Purchased HeLa cervical epithelial cells (Public Health England, Salisbury, UK) and Hep-G2 liver cells (ATCC, Middlesex, UK) were cultured in complete medium comprising DMEM (Thermo Fisher Scientific, Paisley, UK), 10% fetal bovine serum (FBS; Biosera, East Sussex, UK), and 1% antibiotic, antimycotic solution (Merck, Gillingham, UK). The A549 and NCI-H441 lung epithelial cells (both ATCC) were cultured in complete medium comprising RPMI (Thermo Fisher Scientific), 10% FBS, 1% antibiotic, antimycotic solution and 2 mM L-Glutamine (Thermo Fisher Scientific). The FBS used in the present study contained 1.12 ± 0.05 mM cholesterol, as quantified using a cholesterol oxidase-enzymatic assay (Randox Daytona Plus, Randox Laboratories Ltd, Crumlin, UK). Cells were maintained in 75 cm^2^ flasks (Greiner Bio-One) at 37.5⁰C in humidified air with 5% carbon dioxide, with medium replenished every 48 to 72 h. For experiments, 4 × 10^4^ cells/well were seeded in 1 ml/well complete medium using 24-well tissue culture plates (TPP, Trasadingen, Switzerland), and cultured until 70% confluent.

### 2.2 Pore-Forming Toxins

The *plo* plasmid (pGS59) was a gift from Dr H Jost (University of Arizona), and the pyolysin protein was generated and purified as described previously, with a specific activity of 628,338 hemolytic units (HU)/mg protein (21, 30). Streptolysin O was purchased, stored as a 1 mg/ml solution, and activated with 10 mM dithiothreitol according to the manufacturer’s instructions (Merck). *Staphylococcus aureus* α-hemolysin was purchased and stored as a 0.5 mg/ml solution, according to the manufacturer’s instructions (Merck).

### 2.3 Experiments

#### 2.3.1 Cytoprotection

To determine the amount of pyolysin required to study cytoprotection, cells were cultured in serum-free medium for 24 h and then challenged for 2 h with control serum-free medium or medium containing the range of concentrations of pyolysin specified in *Results*. After the challenge, we measured the leakage of LDH into cell supernatants and assessed cytolysis by measuring viable cells using an MTT assay. For subsequent cytoprotection experiments we selected pyolysin challenges that increased LDH leakage and caused cytolysis in HeLa (100 HU/well), A549 (25 HU/well), Hep-G2 (100 HU/well) and NC1-H441 cells (200 HU/well).

To examine whether oxysterols protected against pyolysin, cells were treated in serum-free medium with vehicle (0.1% methanol), 27-hydroxycholesterol, 25-hydroxycholesterol, 7α-hydroxycholesterol, or 7β-hydroxycholesterol (Avanti Polar Lipids, Alabama, United States), using the concentrations and durations specified in *Results*. After the treatment period, supernatants were discarded, and cells were challenged for 2 h, with control serum-free medium or pyolysin, in the absence of further treatment. To determine whether cytoprotection extended beyond pyolysin, HeLa and A549 cells were treated for 24 h with vehicle or 27-hydroxycholesterol, and then challenged with control serum-free medium, or streptolysin O or α−hemolysin as specified in *Results.* The concentration and duration of α-hemolysin challenge was determined by culturing cells for 24 h in serum-free medium and then challenging them for 0 to 24 h using the concentrations specified in *Results*, which were informed by α−hemolysin concentrations used in HeLa cells previously (31). To determine if pyolysin binds directly to oxysterols, pyolysin was mixed with vehicle, 10 ng/ml 27-hydroxycholesterol, or 10 ng/ml 25-hydroxycholesterol, and then used to challenge cells for 2 h. At the end of the cytolysin challenge period, supernatants were collected to measure LDH leakage and viable cells were evaluated by MTT assay or immunofluorescent microscopy.

#### 2.3.2 MAPK Cell Stress Responses

To explore whether oxysterols could protect against pyolysin-induced MAPK activation, 1.5 × 10^5^ HeLa cells/well were cultured in complete medium for 24 h using 6-well plates, and treated in serum-free medium for 24 h with vehicle, 10 ng/ml 27-hydroxycholesterol, or 50 nM T0901317 (Tocris, Abingdon, UK), which is an LXR agonist (32). Cells were then challenge with control serum-free medium or 100 HU pyolysin for 10 min, the supernatants discarded, and the cells washed with 300 μl ice cold PBS and lysed with 100 μl PhosphoSafe Extraction Reagent (Novagen, Darmstadt, Germany) for Western blotting of the MAPK pathway.

#### 2.3.3 Cytoprotection and Ion Flux

To examine the role of potassium efflux or calcium influx on oxysterol protection against pyolysin, HeLa cells were cultured in serum-free medium with vehicle or 10 ng/ml 27-hydroxycholesterol for 24 h. Then, for potassium experiments, pyolysin was prepared in low-potassium medium (5 mM KCl, 140 mM NaCl, 10 mM Hepes, 1.3 mM CaCl_2_, 0.5 mM MgCl_2_, 0.36 mM K_2_HPO_4_, 0.44 mM KH_2_PO_4_, 5.5 mM D-glucose, 4.2 mM NaHCO_3_; Merck) or high-potassium medium (140 mM KCl, 5 mM NaCl, 10 mM Hepes, 1.3 mM CaCl_2_, 0.5 mM MgCl_2_, 0.36 mM K_2_HPO_4_, 0.44 mM KH_2_PO_4_, 5.5 mM D-glucose, 4.2 mM NaHCO_3_) to prevent potassium efflux, as described previously (33). Cells were washed twice with low-potassium or high-potassium medium prior to a 2 h challenge with pyolysin in the cognate medium. For calcium experiments, the cells were washed twice with calcium-free Dulbecco’s phosphate-buffered saline (DPBS, Thermo Fisher Scientific). Cells were then challenged for 2 h with or without pyolysin in control medium (1.8 mM CaCl) or calcium-free medium (Thermo Fisher Scientific) to prevent calcium influx. At the end of the challenge period, supernatants were collected to measure LDH leakage and viable cells were evaluated by MTT assay.

#### 2.3.4 Cytoprotection and Liver X Receptors

To examine the role of LXRα and LXRβ in cytoprotection, we used siRNA to target *NR1H3* and *NR1H2*, prior to treatment with 27-hydroxycholesterol and a subsequent pyolysin challenge. We used the LXR agonist T0901317 as a positive control, with the concentration determined by treating cells with the range of concentrations specified in *Results* and measuring LDH leakage and cell viability following a 2 h pyolysin challenge. Briefly, HeLa cells were transfected with scramble siRNA (ON-TARGETplus Non-targeting Control Pool; Horizon discovery, Cambridge, UK), or siRNA designed using Dharmacon siDesign Centre to target *NR1H3* and *NR1H2* (**Supplementary Table 1**). A mixture of 20 pmol of siRNA, 100 μl Opti-MEM 1 medium and 1.5 µl Lipofectamine RNAiMAX Reagent (both Thermo Fisher Scientific) were added to each well of a 24-well plate and incubated for 20 min, and then 4 × 10^4^ HeLa cells/well were seeded in 900 µl DMEM medium supplemented with 10% FBS for 48 h. Supernatants were discarded and cells were treated with vehicle, 10 ng/ml 27-hydroxycholesterol or 50 nM T0901317 for 24 h in serum-free culture medium prior to a 2 h challenge with control medium or pyolysin. At the end of the challenge period, supernatants were collected to measure LDH leakage and cell viability was evaluated with MTT assay.

#### 2.3.5 Cytoprotection and Cholesterol

To investigate the effect of oxysterol treatment on pyolysin binding to accessible cholesterol in the cell membrane, 1.5 × 10^5^ HeLa cells/well were cultured in complete medium for 24 h using 6-well plates, and then treated in serum-free medium with vehicle, 10 ng/ml 27-hydroxycholesterol or 50 nM T0901317 for 24 h, before a 2 h challenge with control serum-free medium or pyolysin. The cells were washed with 300 μl ice cold PBS and lysed with 100 μl PhosphoSafe Extraction Reagent for Western blotting for pyolysin binding.

To examine the role of serum cholesterol in cytoprotection against pyolysin we first cultured HeLa cells for 24 h in medium containing 10% serum with vehicle or 25-hydroxycholesterol, followed by challenge with control medium or pyolysin for 2 h. To verify protection against pyolysin by statins inhibiting HMGCR (34), HeLa cells were cultured for 24 h in serum-free medium with vehicle or 10 µM atorvastatin (Merck), followed by challenge with control medium or pyolysin for 2 h. To examine the role of ACAT esterification of cholesterol in cytoprotection against pyolysin, we used the selective ACAT inhibitor Sandoz 58-035 (SZ58-035, Merck), as described previously (10, 35). Cells were washed twice with phosphate-buffered saline (PBS, Thermo Fisher Scientific), and cultured in serum-free medium with vehicle (0.1% DMSO) or 10 µM SZ58-035 for 16 h. The cells were then washed twice with PBS, and treated with vehicle, 10 ng/ml 27-hydroxycholesterol or 10 ng/ml 25−hydroxycholesterol for 24 h in the medium containing DMSO or SZ58-035, followed by challenge with control medium or pyolysin, for 2 h. At the end of the challenge periods, supernatants were collected to measure LDH leakage, and cell viability was measured using an MTT assay.

To explore the effect of increasing membrane accessible cholesterol on protection against pyolysin, sphingomyelin-cholesterol complexes were disrupted using sphingomyelinase from *Staphylococcus aureus* (Merck), as described previously (5, 10). Cells were treated in serum-free medium with vehicle (0.1% methanol) or 10 ng/ml 27-hydroxycholesterol for 24 h. The cells were washed twice with PBS and cultured in serum-free medium with or without 100 mU/ml sphingomyelinase for 30 min, washed twice with PBS, and then challenged with control medium or 25 HU pyolysin for 2 h. In preliminary experiments, we titrated the amount of pyolysin used to challenge the cells because the cells were more susceptible to pyolysin following sphingomyelinase treatment, with 100 HU pyolysin causing excessive cell lysis (> 97%), as determined by MTT assay. At the end of the challenge periods, supernatants were collected to measure LDH leakage, and cell viability was measured using an MTT assay.

### 2.4 Cell Viability

The viability of cells was assessed by the mitochondria-dependent reduction of 3-(4,5-dimethylthiazol-2-yl)-2,5-diphenyltetrazolium bromide (MTT, Merck) as described previously (21). Briefly, cells were incubated in 250 µl/well of serum-free medium containing 1 mg/ml MTT for 2 h, the medium was then discarded, and cells were lysed with 300 µl of dimethyl sulfoxide (Merck). Optical density (OD_570_) was measured using a POLARstar Omega micro plate reader (POLARstar Omega; BMG, Labtech, Ortenberg, Germany).

### 2.5 Lactate Dehydrogenase

Cell supernatant LDH was quantified by LDH−dependent conversion of lactate to pyruvate, *via* reduction of β−Nicotinamide adenine dinucleotide sodium salt (NAD+) to NADH, which is detected by NADH−dependent reduction of a tetrazolium salt to formazan, as described previously (18). Inter− and intra−assay coefficients of variation were < 4%.

### 2.6 Potassium

The leakage of potassium was measured as described previously (17, 29). Briefly, 1.5 × 10^5^ cells/well were cultured for 24 h in complete medium using 6-well culture plates, and then treated with 27-hydroxycholesterol or 25-hydroxycholesterol for 24 h using the concentrations specified in *Results*. The cells were washed and challenged in potassium-free buffer for 5 min with the amounts of pyolysin or streptolysin O indicated in *Results*, or for 15 min with 8 µg α-hemolysin (no potassium leakage was detected after 5 min of α-hemolysin challenge). Extracellular potassium was measured in cell supernatants using a Jenway PFP7 flame photometer (Cole-Parmer, Stone, Staffordshire, UK). The inter- and intra-assay coefficients of variation were < 4%.

### 2.7 Cholesterol

To measure cellular cholesterol, 1.5 × 10^5^ HeLa cells/well were cultured in complete medium for 24 h using 6-well culture plates (TPP), and then treated in serum-free medium with 10 ng/ml 27-hydroxycholesterol, 10 ng/ml 25-hydroxycholesterol, 1 mM methyl-β-cyclodextrin (Merck) or 10 µM atorvastatin for 24 h. The cells were then washed twice with PBS, collected in 200 µl/well cholesterol assay buffer (Thermo Fisher Scientific), and stored at -20⁰C. Cellular cholesterol was measured using the Amplex Red Cholesterol Assay Kit (Thermo Fisher Scientific) according to the manufacturer’s instructions. Cholesterol concentrations were normalized to total protein concentrations, measured using a DC protein assay (Bio-Rad Laboratories, Hercules, CA, United States), as described previously (18, 29). The inter- and intra-assay coefficients of variation for the cholesterol assay were < 5% and < 6% respectively.

### 2.8 Immunofluorescent Microscopy

We stained cells using phalloidin to visualize cytoskeletal and cell damage, as described previously (17). Briefly, 4 × 10^4^ cells/well were cultured on glass cover slips in complete-medium for 24 h using 24-well culture plates, and then treated for 24 h in serum-free medium with vehicle, 27-hydroxycholesterol, 25-hydroxycholesterol or T0901317 as specified in *Results*. The cells were then challenged with control medium or pyolysin for 2 h, or α-hemolysin for 24 h. At the end of the challenge period, cells were washed with PBS, fixed with 4% paraformaldehyde (Merck), washed with PBS, and permeabilized in 0.2% Triton-X 100. The cells were then blocked for 30 min in PBS containing 0.5% bovine serum albumin (BSA; Merck) and 0.1% Triton-X 100, and then incubated for 1 h with Alexa Fluor 555-conjugated phalloidin (Thermo Fisher Scientific). Cells were washed with 0.1% Triton-X 100 in PBS three times and mounted onto microscope slides using 40,6 diamidino-2-phenylindole to visualize cell nuclei (Vectashield with DAPI; Vector Laboratories Inc., Burlington, CA, USA). The cells were examined using an Axio Imager M1 fluorescence microscope and images captured using an AxioCamMR3 (Zeiss, Jena, Germany). The proportion of cells that had cytoskeletal changes (cytoskeletal contraction, disrupted shape, or loss of actin fiber definition) were counted using > 135 cells per treatment across 3 independent images per replicate.

### 2.9 Quantitative PCR

Total RNA was isolated from HeLa cells lysed in RLT buffer using the RNeasy Mini Kit according to the manufacturer’s instructions (QIAGEN, Crawley, UK). The RNA was quantified with a Nanodrop spectrophotometer (Labtech, Ringmer, UK), and reverse transcription of 1 µg mRNA was performed using the Quantitect Reverse Transcription Kit according to the manufacturer’s instructions (QIAGEN). Primers were designed using the NCBI primer design tool for *NR1H2*, *NR1H3* and the reference gene ribosome-like protein 19 (*RPL19*; **Supplementary Table 2**). Quantitative PCR was performed in 25 µl reaction volumes comprising 12.5 µl QuantiFast SYBR Green PCR Master Mix (QIAGEN), 0.25 µL forward primer, 0.25 µl reverse primer, 10.5 µl nuclease-free water, and 1.5 μl cDNA on white low-profile 96-well plates (Bio-Rad Laboratories). Thermal cycling parameters were a cycle of 95°C for 5 min, followed by 40 cycles of 95°C for 10 s and 60°C for 30 s, using the CFX Connect Real-time thermal cycler (Bio-Rad Laboratories). Gene expression analysis was performed according to the MIQE guidelines (36). Standard curves were generated from serial dilutions of mRNA extracted from untreated HeLa cells. Gene expression was analyzed in triplicate using the appropriate standard curve quantification cycle, and mRNA expression normalized to *RPL19*.

### 2.10 Western Blotting

Protein isolation and Western blotting was performed as described previously (30, 37). Protein was extracted and quantified by DC assay, and 10 μg/lane protein separated using 10% (vol/vol) SDS polyacrylamide gel electrophoresis. Proteins were transferred onto a polyvinylidene difluoride membrane (GE Healthcare, Chalfont, St Giles, UK), and blocked for 1 h in Tris buffered saline 0.1% Tween 20 (TBST, Merck) with 5% BSA. The membranes were then incubated overnight at 4⁰C in TBST 5% BSA with 1:1000 diphosphorylated ERK1/2 (Research Resource Identifier, RRID: AB_477245; Merck), ERK1/2 (RRID: AB_2297336; Abcam, Cambridge, UK), phospho-p38 (RRID: AB_2139682, Cell Signaling, Danvers, MA, USA), p38 (RRID: AB_10999090, Cell Signaling), phospho-JNK (RRID: AB_331659; Cell Signaling), JNK (RRID: AB_2250373; Cell Signaling), α-tubulin (RRID: AB_2210548; Cell Signaling), or 1:500 anti-pyolysin antibody (19). Membranes were washed five times in TBST, and then incubated in TBST 5% BSA for 1 h at room temperature with 1:2500 anti-rabbit IgG (RRID: AB_2099233; Cell Signaling) or anti-mouse IgG (RRID: AB_330924; Cell Signaling). Membranes were washed a further five times in TBST, and protein reactivity was visualized using enhanced chemiluminescence (Clarity Western ECL substrate, Bio-Rad Laboratories). Membrane images were captured using a ChemiDoc XRS System (Bio-Rad Laboratories), and the average peak band density quantified and normalized to α-tubulin using Fiji, as described previously (29, 38).

### 2.11 Statistical Analysis

The statistical unit was each independent passage of cells. Statistical analysis was performed using GraphPad Prism 9.0.1 (GraphPad Software, San Diego, California, USA), with significance attributed when P < 0.05, and data presented as arithmetic mean ± s.e.m. Comparisons between treatments were made using independent t-test, or using ANOVA followed by Dunnett, Bonferroni or Tukey *post hoc* test for multiple comparisons, as specified in *Results*.

## 3 Results

### 3.1 Oxysterols Protect HeLa Cells Against Pyolysin

We first determined the amount of pyolysin required to damage HeLa cells. We used HeLa cells because these epithelial cells are often used to explore responses to cholesterol-dependent cytolysins (14, 18, 24, 33). Although culturing cells in medium containing 10% serum did not significantly alter pyolysin-induced leakage of LDH or cytolysis compared with serum-free medium (**Supplementary Figure 1**), we used serum-free medium to avoid pyolysin binding to serum cholesterol, or serum altering cholesterol homeostasis or inducing MAPK phosphorylation (39, 40). We used a 2 h challenge because we aimed to examine whether oxysterols stimulated the intrinsic protection of cells against damage caused by cytolysins, whereas a longer challenge might also reflect membrane repair and cell replication (16, 21, 30). Pyolysin caused pore formation as determined by the leakage of LDH into supernatants from HeLa cells cultured in 24-well plates, and caused cytolysis as determined by reduced cell viability (**Supplementary Figure 2A**). For subsequent cytoprotection experiments, we used 100 HU pyolysin per well because this reduced HeLa cell viability by > 80%.

**Figure 1:**
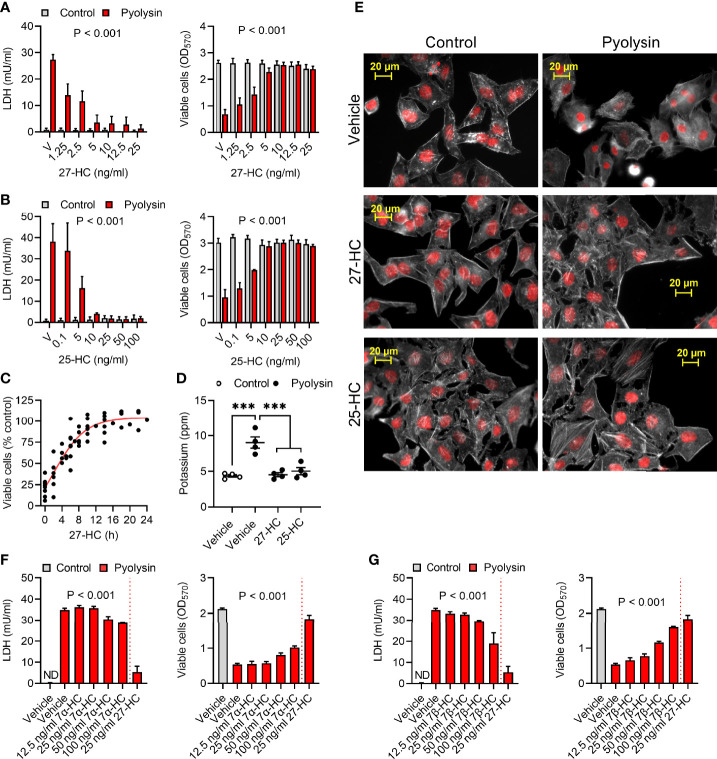
Oxysterols protect HeLa cells against pyolysin. **(A, B)** HeLa cells were cultured for 24 h in serum-free medium with vehicle (V) or the indicated concentrations of **(A)** 27-hydroxycholesterol (27-HC) or **(B)** 25-hydroxycholesterol (25-HC) and then challenged for 2 h with control serum-free medium (■) or 100 HU pyolysin (■). Leakage of LDH was measured in cell supernatants, and viable cells were determined by MTT assay. Data are presented as mean + s.e.m. from 7 **(A)** or 3 **(B)** independent experiments; data were analyzed by ANOVA and P values reported for the treatment effect on pyolysin challenge. **(C)** HeLa cells were cultured for the indicated times in serum-free medium with 10 ng/ml 27-HC, challenged for 2 h with control serum-free medium or 100 HU pyolysin, and viable cells were determined by MTT assay. Pyolysin data are presented as percentage of control challenge; dots represent independent measurements across 5 experiments; the line is the least squares fit. **(D)** Leakage of potassium from HeLa cells cultured for 24 h in serum-free medium with vehicle, 10 ng/ml 27-HC or 10 ng/ml 25-HC, and then challenged for 5 min with control serum-free medium (○) or 100 HU pyolysin (●). Extracellular potassium was determined in cell supernatants by flame photometry. Data are presented as mean ± s.e.m. with dots representing values from 4 independent experiments; data were analyzed by ANOVA with Dunnett’s *post hoc* test, ***P < 0.001. **(E)** Fluorescent microscope images of HeLa cells cultured for 24 h in serum-free medium with vehicle, 10 ng/ml 27-HC or 10 ng/ml 25-HC, and then challenged for 2 h with control serum-free medium or pyolysin. Cells were stained with Alexa Fluor 555-conjugated phalloidin to visualize F-actin (white) and fluorescent microscope images collected (nuclei are red); images are representative of 3 experiments. **(F, G)** HeLa cells were cultured for 24 h in serum-free medium with vehicle or the indicated concentrations of 7α-hydroxycholesterol (7α-HC, F), 7β-hydroxycholesterol (7β-HC, G) or 27-HC and then challenged for 2 h with control serum-free medium (■) or 100 HU pyolysin (■). The leakage of LDH was measured in cell supernatants, and viable cells were determined by MTT assay. Data are presented as mean + s.e.m. from 4 independent experiments; data were analyzed by ANOVA and P values reported for the effect of 7α-hydroxycholesterol or 7β-hydroxycholesterol on pyolysin challenge.

We next investigated whether treatment with the side-chain hydroxycholesterols 27-hydroxycholesterol or 25-hydroxycholesterol protected HeLa cells against a subsequent pyolysin challenge. The cells were treated for 24 h with a range of oxysterol concentrations, informed by human plasma concentrations (41), and then challenged with pyolysin for 2 h without further oxysterol treatment. Both 27-hydroxycholesterol and 25-hydroxycholesterol reduced pyolysin-induced leakage of LDH and cytolysis (**Figures 1A, B**). Specifically, treatment with 10 ng/ml 27-hydroxycholesterol for 24 h reduced LDH leakage by 88% when cells were subsequently challenged with pyolysin for 2 h, and reduced cytolysis from 74% to 1%, whilst 10 ng/ml 25-hydroxycholesterol reduced pyolysin-induced LDH leakage by 89% and cytolysis from 68% to 2%.

Cytoprotection was evident after 4 h treatment with 27-hydroxycholesterol and was maximal by 16 to 24 h (**Figure 1C**, P < 0.001, ANOVA and Dunnett’s *post hoc* test, n = 5). Treating cells with 10 ng/ml 27-hydroxycholesterol or 25-hydroxycholesterol for 24 h also prevented leakage of potassium following a subsequent 5 min pyolysin challenge (**Figure 1D**). Furthermore, compared with the pyolysin-induced reduction in actin fiber definition and cytoskeletal collapse in vehicle treated cells, treatment with 27-hydroxycholesterol or 25-hydroxycholesterol limited cytoskeletal changes (83 ± 5% vs 18 ± 5% or 20 ± 4% cells damaged, ANOVA with Dunnett’s *post hoc* test, n = 3 independent experiments, P < 0.001; **Figure 1E**).

We also examined whether treatment with ring-modified hydroxycholesterols for 24 h might protect HeLa cells against a subsequent 2 h pyolysin challenge. Both 7α-hydroxycholesterol and 7β-hydroxycholesterol reduced pyolysin-induced LDH leakage and cytolysis, but neither were as effective as an equivalent concentration of 27-hydroxycholesterol (**Figures 1F, G**). Collectively these data provide evidence that oxysterols protect HeLa cells against damage caused by pyolysin. As side-chain hydroxycholesterols were most protective, and 27-hydroxycholesterol is abundant in human plasma (41), we focused on their ability to stimulate the intrinsic protection of epithelial cells against cytolysins.

### 3.2 Side-Chain Hydroxycholesterols Protect Epithelial Cells Against Pyolysin

**Figure 2:**
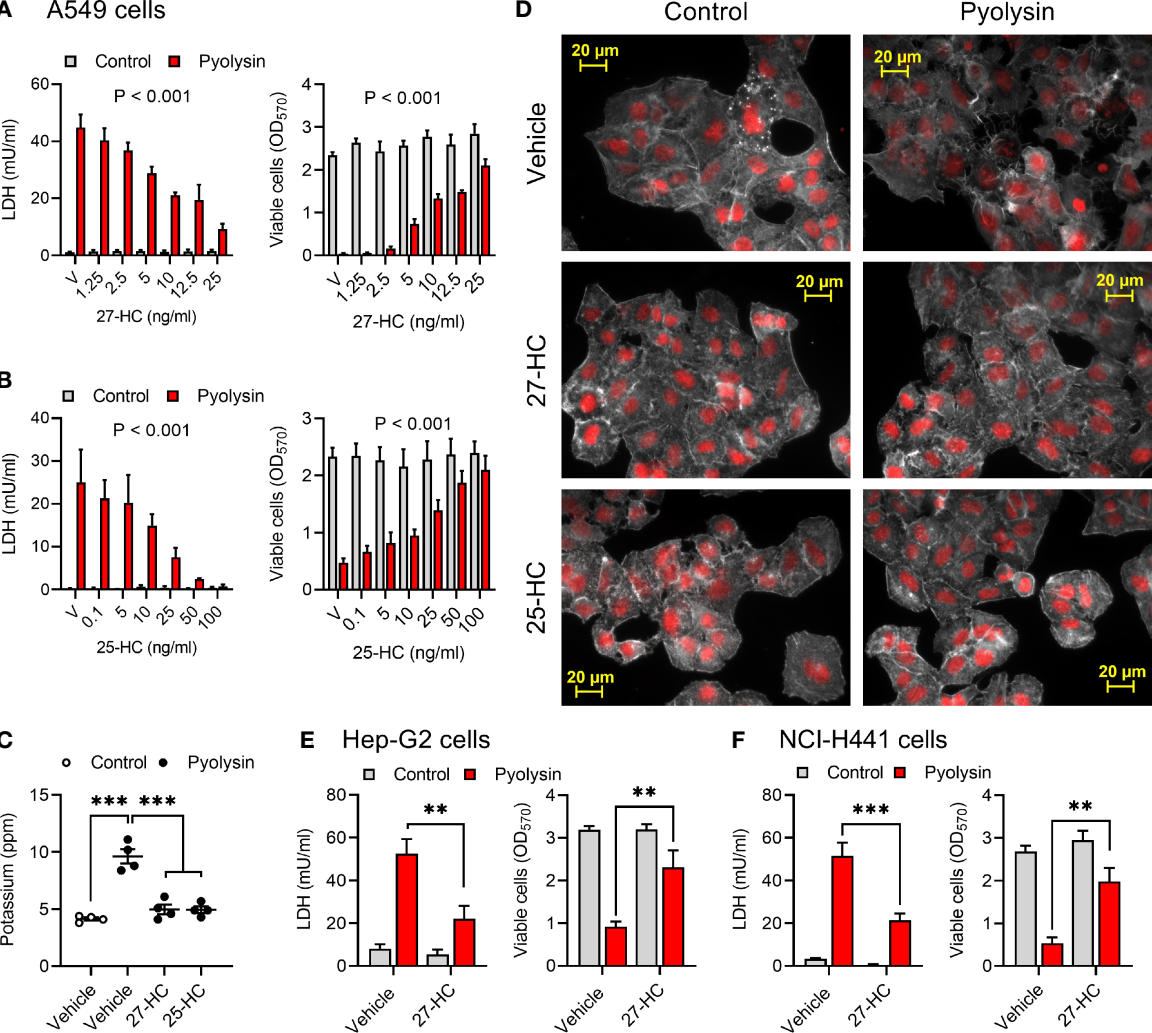
Side-chain hydroxycholesterols protect epithelial cells against pyolysin. **(A, B)** A549 cells were cultured for 24 h in serum-free medium with vehicle (V) or the indicated concentrations of **(A)** 27-hydroxycholesterol or **(B)** 25-hydroxycholesterol, and then challenged for 2 h with control serum-free medium (■) or 25 HU pyolysin (■). The leakage of LDH was measured in cell supernatants and viable cells were determined by MTT assay. Data are presented as mean + s.e.m. from 4 independent experiments. Data were analyzed by ANOVA and P values reported for the treatment effect on pyolysin challenge. **(C)** Leakage of potassium from A549 cells cultured for 24 h in serum-free medium with vehicle, 25 ng/ml 27-hydroxycholesterol or 50 ng/ml 25-hydroxycholesterol, and then challenged for 5 min with control serum-free medium (○) or 25 HU pyolysin (●). Extracellular potassium was measured in supernatants by flame photometry. Data are presented as mean ± s.e.m. with dots representing values from 4 independent experiments; data were analyzed by ANOVA with Dunnett’s *post hoc* test, ***P < 0.001. **(D)** Fluorescent microscope images of A549 cells cultured for 24 h in serum-free medium with vehicle, 10 ng/ml 27-HC or 10 ng/ml 25-HC, and then challenged for 2 h with control serum-free medium or pyolysin. Cells were stained with Alexa Fluor 555-conjugated phalloidin to visualize F-actin (white) and fluorescent microscope images collected (nuclei are red, scale bars are 20 μm); images are representative of 3 experiments. **(E)** Hep-G2 liver cells and **(F)** NCI-H441 normal lung cells were cultured for 24 h in serum-free medium with vehicle or 10 ng/ml 27-hydroxycholesterol, and then challenged for 2 h control serum-free medium (■) or pyolysin (■; Hep-G2, 100 HU; NCI-H441, 200 HU). The leakage of LDH was measured in cell supernatants and viable cells were determined by MTT assay. Data were analyzed by ANOVA with Tukey’s *post hoc* test, ***P < 0.001,**P < 0.01.

To examine if oxysterol cytoprotection extended beyond HeLa cells, we used A549 lung alveolar epithelial cells as they are used to explore responses to cytolysins (42, 43). Challenging A549 cells with 25 HU/well pyolysin for 2 h reduced A549 cell viability by > 80% (**Supplementary Figure 2B**). However, treatment with 27-hydroxycholesterol or 25-hydroxycholesterol for 24 h reduced pyolysin-induced leakage of LDH and cytolysis (**Figures 2A, B**). Specifically, 25 ng/ml 27-hydroxycholesterol or 50 ng/ml 25-hydroxycholesterol reduced pyolysin-induced LDH leakage by 80% and 91%, respectively, and reduced cytolysis from ≥ 80% to < 27%. Both hydroxycholesterols also prevented leakage of potassium induced by a 5 min pyolysin challenge (**Figure 2C**). Furthermore, compared with vehicle treated cells, 27-hydroxycholesterol and 25-hydroxycholesterol reduced pyolysin-induced cytoskeletal changes (73 ± 3% vs 17 ± 3% and 15 ± 5% cells damaged, ANOVA with Dunnett’s *post hoc* test, n = 3, P < 0.001; **Figure 2D**).

We also examined cytoprotection using Hep-G2 liver epithelial cells and NCI-H441 normal lung epithelial cells. Pyolysin caused LDH leakage and cytolysis in Hep-G2 and NCI-H441 cells (**Supplementary Figures 2C, D**). However, treatment with 10 ng/ml 27-hydroxycholesterol for 24 h reduced pyolysin-induced LDH leakage by 58% and 59% in Hep-G2 and NCI-H441 cells, respectively, and reduced pyolysin-induced cytolysis from > 70% to < 30% (**Figures 2E, F**). These data imply that side-chain hydroxycholesterols protect multiple epithelial cell types against pyolysin.

### 3.3 27-Hydroxycholesterol Prevents MAPK Phosphorylation

**Figure 3:**
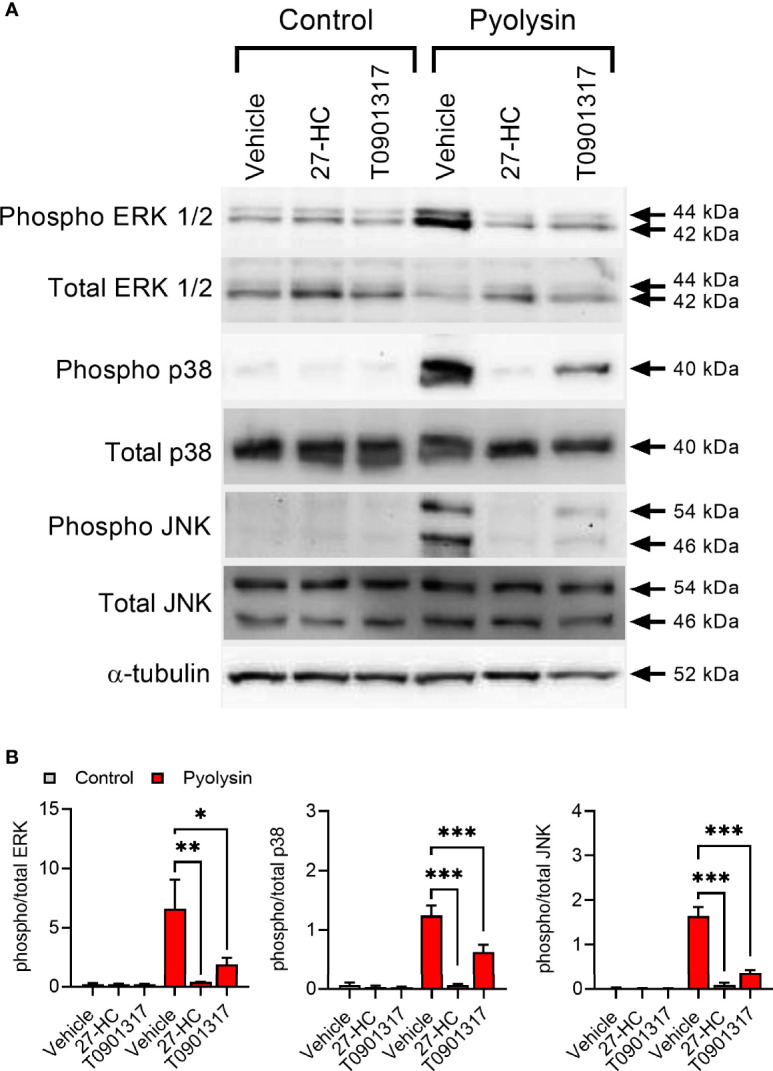
27-hydroxycholesterol prevents MAPK phosphorylation. **(A)** Representative Western blots of phosphorylated and total ERK1/2, p38 and JNK, and α-tubulin for HeLa cells treated with vehicle, 10 ng/ml 27-hydroxycholesterol (27-HC) or 50 nM T0901317 for 24 h, and challenged with control serum-free medium or 100 HU pyolysin for 10 min. **(B)** Densitometry data were normalized to α-tubulin, and presented as mean + s.e.m. from 3 independent experiments; statistical significance was determined using ANOVA with Dunnett’s *post hoc* test, ***P < 0.001, **P < 0.01, *P < 0.05.

Pyolysin induces a MAPK cell stress response, including pyolysin-induced phosphorylation of ERK, JNK and p38 in HeLa cells (18, 24). Treatment with 27-hydroxycholesterol reduced pyolysin-induced ERK 1/2, p38 and JNK phosphorylation (**Figures 3A, B** and **Supplementary Figure 3**). The reduction in pyolysin-induced MAPK phosphorylation with 27-hydroxycholesterol was as effective as treatment with an LXR agonist (T0901317), which was used as a positive control because LXR agonists protect bovine endometrial cells and murine macrophages against cholesterol-dependent cytolysins (28, 29). These data provide evidence that 27-hydroxycholesterol prevents activation of MAPK cell stress responses to pyolysin.

### 3.4 27-Hydroxycholesterol Protects Cells Against Streptolysin O and α-Hemolysin

**Figure 4:**
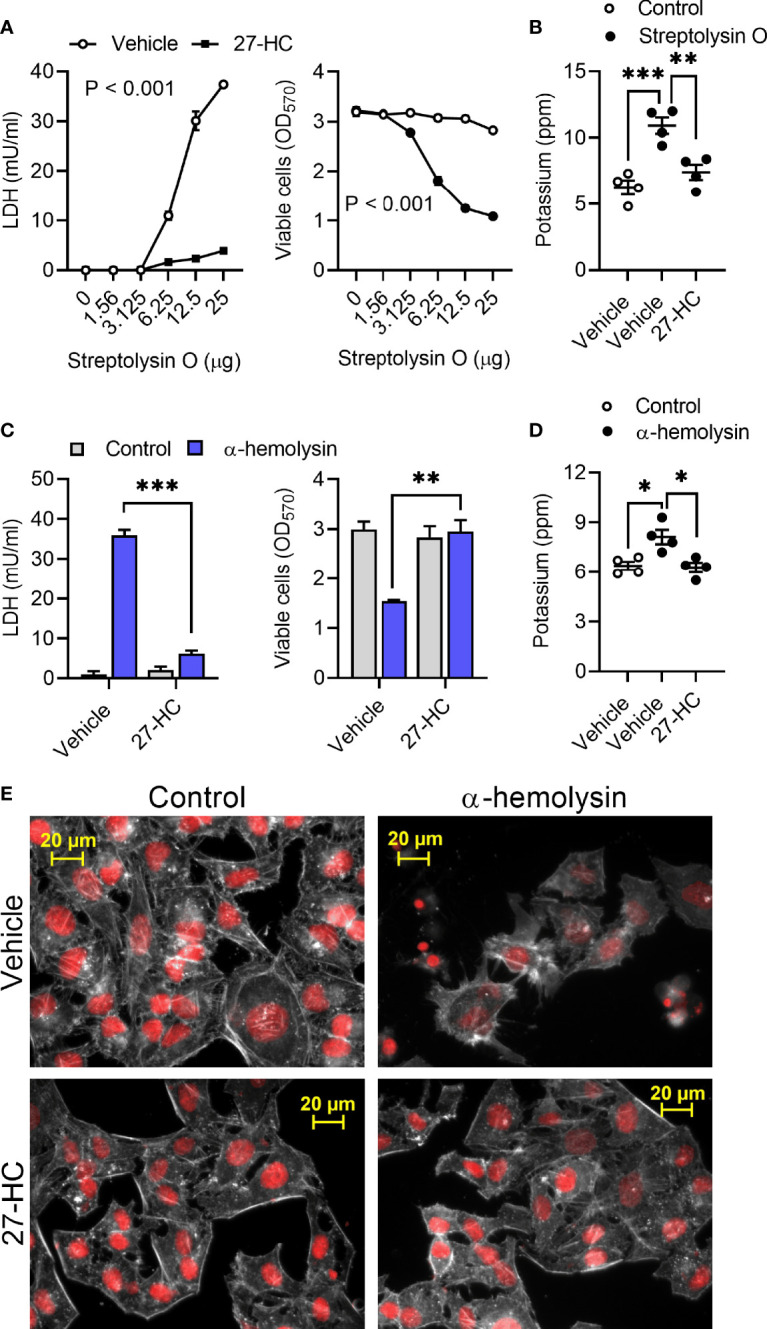
27- hydroxycholesterol protects cells against streptolysin O and α-hemolysin. **(A)** HeLa cells were cultured for 24 h in serum-free medium with vehicle (○) or 25 ng/ml 27-hydroxycholesterol (27-HC, ●), and then challenged for 24 h with the indicated concentrations of streptolysin O. The leakage of LDH was measured in cell supernatants and viable cells were determined by MTT assay. Data are presented as mean ± s.e.m. from 4 independent experiments. Data were analyzed by ANOVA. **(B)** The leakage of potassium from HeLa cells cultured for 24 h in serum-free medium with vehicle or 25 ng/ml 27-HC, and then challenged for 5 min with control serum-free medium (○) or streptolysin O (●). Extracellular potassium was measured in supernatants by flame photometry. Data are presented as mean ± s.e.m. with dots representing the values from 4 independent experiments. Data were analyzed by ANOVA with Dunnett’s *post hoc* test, ***P < 0.001, **P < 0.01. **(C)** HeLa cells were cultured for 24 h in serum-free medium with vehicle or 25 ng/ml 27-HC, and then challenged for 24 h with control medium (■) or 8 µg/well α-hemolysin (■). The leakage of LDH was measured in cell supernatants and viable cells were determined by MTT assay. Data were analyzed by ANOVA with Tukey’s *post hoc* test, ***P < 0.001, **P < 0.01. **(D)** The leakage of potassium from A549 cells cultured for 24 h in serum-free medium with vehicle or 25 ng/ml 27-HC, and then challenged for 15 min with control serum-free medium (○) or 8 µg/well α-hemolysin (●). Extracellular potassium was measured in supernatants by flame photometry. Data are presented as mean ± s.e.m. with dots representing the values from 4 independent experiments. Data were analyzed by ANOVA with Dunnett’s *post hoc* test, *P < 0.05. **(E)** Fluorescent microscope images of HeLa cells cultured for 24 h in serum-free medium with vehicle or 10 ng/ml 27-HC, and then challenged for 2 h with control serum-free medium or α-hemolysin. Cells were stained with Alexa Fluor 555-conjugated phalloidin to visualize F-actin (white) and fluorescent microscope images collected (nuclei are red); images are representative of 3 experiments.

To explore whether 27-hydroxycholesterol cytoprotection extended beyond pyolysin we used *Streptococcus pyogenes* streptolysin O and *Staphylococcus aureus* α-hemolysin. Streptolysin O is a cholesterol-dependent cytolysin that forms 26 nm diameter β-barrel transmembrane pores, damages cells and causes cytolysis (44, 45). Challenging HeLa cells with Streptolysin O for 2 h caused pore formation and cytolysis as expected, but treatment with 25 ng/ml 27-hydrocycholesterol for 24 h reduced streptolysin-induced leakage of LDH, cytolysis, and leakage of potassium (**Figures 4A, B**).

*Staphylococcus aureus* α-hemolysin forms 1.4 nm internal-diameter β-barrel transmembrane pores that cause cytolysis (46, 47). Challenging HeLa or A549 cells with 8 µg/well α-hemolysin for 24 h caused pore formation and cytolysis (**Supplementary Figure 4**). However, treatment with 25 ng/ml 27-hydrocycholesterol for 24 h reduced the α-hemolysin-induced leakage of LDH, cytolysis, and leakage of potassium in HeLa cells (**Figures 4C, D**) and in A549 cells (**Supplementary Figures 5A, B**). Compared with vehicle, treatment with 27-hydroxycholesterol also reduced α-hemolysin-induced cytoskeletal changes in HeLa cells (85 ± 3% vs 35 ± 1% cells damaged, t-test, n = 3, P < 0.001; **Figure 4E**) and A549 cells (84 ± 6% vs 19 ± 4% cells damaged, t-test, n = 3, P < 0.01; **Supplementary Figure 5C**). These data provide evidence that 27-hydrocycholesterol protects epithelial cells against an α-hemolysin, as well as protecting against cholesterol-dependent cytolysins.

### 3.5 Potassium and Calcium Do Not Affect 27-Hydroxycholesterol Cytoprotection

**Figure 5:**
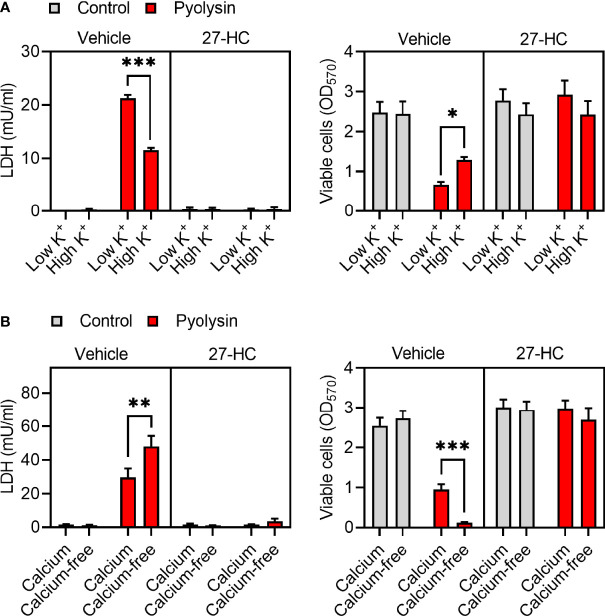
Potassium and calcium do not affect 27-hydroxycholesterol cytoprotection. HeLa cells were cultured for 24 h in serum-free medium with vehicle or 10 ng/ml 27-hydroxycholesterol (27-HC), and then challenged with control medium (■) or 100 HU pyolysin (■) for 2 h in **(A)** low or high-potassium medium, or **(B)** in calcium-containing or calcium-free medium. The leakage of LDH was measured in cell supernatants and viable cells assessed by MTT assay. Data are presented as mean + s.e.m. with dots representing the values of cells from 4 independent experiments; statistical significance was determined using ANOVA with Tukey’s *post hoc* test, ***P < 0.001, **P < 0.01, *P < 0.05.

We next investigated potential mechanisms of side-chain hydroxycholesterol cytoprotection against pyolysin. Oxysterols have pleiotropic effects, including altering ion fluxes across cell membranes, increasing MAPK phosphorylation, activating LXRs, accelerating HMGCR degradation, and stimulating ACAT cholesterol esterification (7, 10, 48, 49). We first considered whether oxysterol cytoprotection was dependent on potassium or calcium ion fluxes because these are important for activating cell repair mechanisms (13, 16). Challenging HeLa cells with pyolysin in 140 mM high-potassium medium to prevent potassium efflux resulted in reduced LDH leakage and cytolysis compared with challenging cells in a 5 mM low-potassium medium, but the cytoprotective effect of 27-hydroxycholesterol was unaffected (**Figure 5A**). Challenging HeLa cells with pyolysin in calcium-free medium to prevent calcium influx resulted in increased LDH leakage and cytolysis, but the cytoprotective effect of 27-hydroxycholesterol was not significantly affected (**Figure 5B**).

### 3.6 Liver X Receptors Are Not Required for 27-Hydroxycholesterol Cytoprotection

**Figure 6:**
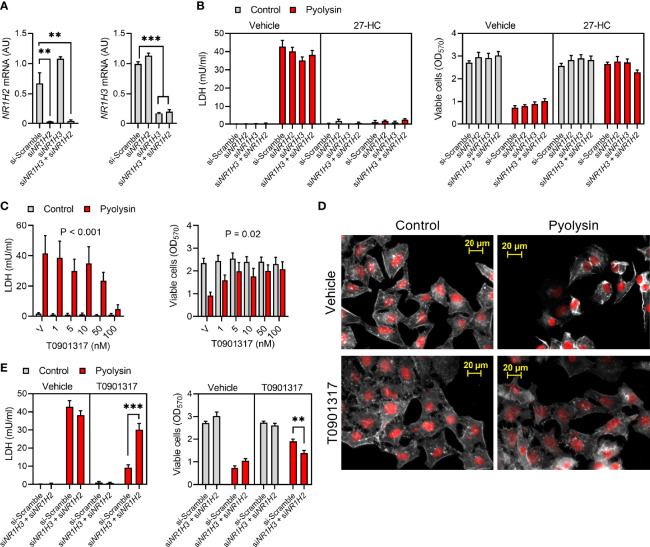
Liver X receptors are not required for 27-hydroxycholesterol cytoprotection. **(A)** Normalized *NR1H2* and *NR1H3* mRNA expression measured by qPCR for HeLa cells transfected with scramble siRNA or siRNA targeting *NR1H3*, *NR1H2*, or both *NR1H3* and *NR1H2*. Data are presented as mean + s.e.m. from 3 independent experiments; data were analyzed by ANOVA with Dunnett’s *post hoc* test, ***P < 0.001, **P < 0.01. **(B)** HeLa cells were transfected for 48 h with scramble siRNA or siRNA targeting *NR1H3*, *NR1H2*, or both *NR1H3* and *NR1H2*; cultured for 24 h in medium with vehicle or 10 ng/ml 27-hydroxycholesterol (27-HC); and, then challenged for 2 h with control serum-free medium (■) or 100 HU pyolysin (■). Leakage of LDH was measured in cell supernatants, and viable cells were determined by MTT assay. Data are presented as mean + s.e.m. from 4 independent experiments. **(C)** HeLa cells were cultured for 24 h in serum-free medium with vehicle (V) or the indicated concentrations of T0901317, and then challenged for 2 h with control serum-free medium (■) or 100 HU pyolysin (■). The leakage of LDH was measured in cell supernatants and viable cells were determined by MTT assay. Data are presented as mean + s.e.m. from 4 independent experiments; data were analyzed by ANOVA and P values reported for the treatment effect on pyolysin challenge. **(D)** Fluorescent microscope images of HeLa cells cultured for 24 h in serum-free medium with vehicle or 50 nM T0901317, and then challenged for 2 h with control medium or 100 HU pyolysin. Cells were stained with Alexa Fluor 555-conjugated phalloidin to visualize F-actin (white) and fluorescent microscope images collected (nuclei are red); images are representative of 3 experiments. **(E)** HeLa cells were transfected for 48 h with scramble siRNA or siRNA targeting both *NR1H3* and *NR1H2*; cultured for 24 h in medium with vehicle or 50 nM T0901317; and, then challenged for 2 h with control serum-free medium (■) or 100 HU pyolysin (■). Leakage of LDH was measured in cell supernatants, and viable cells were determined by MTT assay. Data are presented as mean + s.e.m. from 4 independent experiments; statistical significance was determined by ANOVA and Tukey’s *post hoc* test, ***P < 0.001, **P < 0.01.

Oxysterols such as 27-hydroxycholesterol activate LXRα and LXRβ, encoded by *NR1H3* and *NR1H2*, respectively (50). To test whether 27-hydroxycholesterol cytoprotection was dependent on LXRs, we transfected HeLa cells with siRNA targeting *NR1H3* and *NR1H2*, which reduced expression by > 80% (**Figure 6A**). However, siRNA targeting *NR1H3*, *NR1H2*, or *NR1H3* and *NR1H2* in combination, did not significantly diminish 27-hydroxycholesterol cytoprotection against pyolysin, compared with cells treated with scramble siRNA (**Figure 6B**). To verify that the siRNA was effective we used the LXRα and LXRβ agonist T0901317 (32). We first established that treating HeLa cells with 50 nM T0901317 for 24 h reduced pyolysin-induced leakage of LDH and cytolysis (**Figure 6C**). Furthermore, compared with vehicle, treatment with T0901317 limited pyolysin-induced changes in cell shape (83 ± 5% vs 37 ± 9% cells damaged, t-test, n = 3, P < 0.05, **Figure 6D**). We then confirmed that siRNA targeting *NR1H3* and *NR1H2* diminished the protective effects of T0901317 (**Figure 6E**). These data provide evidence that LXR activation protected cells against pyolysin, but that this was not a mechanism for 27-hydroxycholesterol cytoprotection.

### 3.7 Oxysterols Reduce Accessible Cholesterol

**Figure 7:**
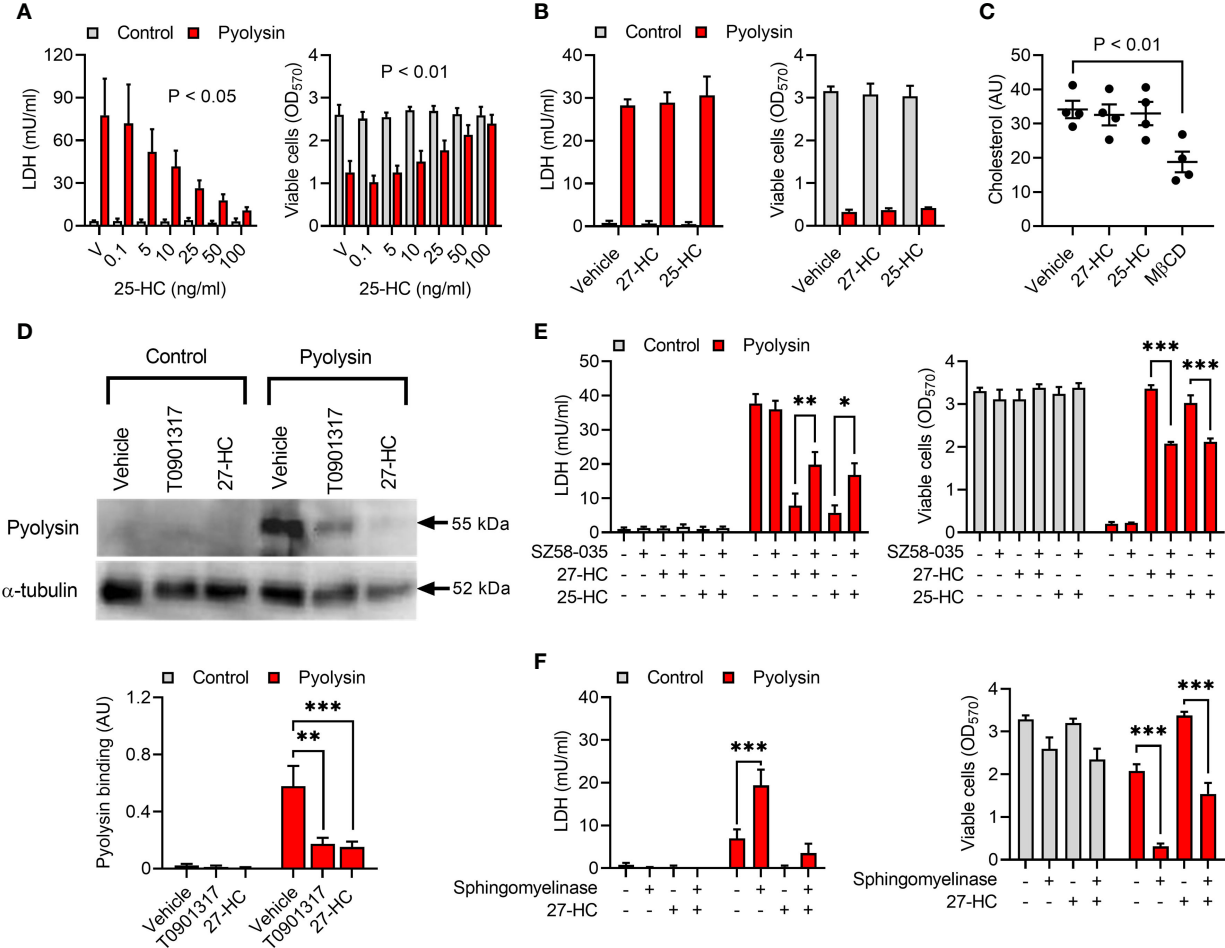
Oxysterols reduce accessible cholesterol. **(A)** HeLa cells were cultured for 24 h in medium containing 10% serum with vehicle (V) or the indicated concentrations of 25-hydroxycholesterol (25-HC), and then challenged for 2 h with control serum-free medium (■) or 100 HU pyolysin (■). The leakage of LDH was measured in cell supernatants and viable cells were determined by MTT assay. Data are presented as mean + s.e.m. from 4 independent experiments; statistical significance was determined by ANOVA, and P values reported for the treatment effect on pyolysin challenge. **(B)** HeLa cells were challenged for 2 h with control serum-free medium (■) or 100 HU pyolysin (■) mixed with vehicle, 10 ng/ml 27-hydroxycholesterol (27-HC), or 10 ng/ml 25-HC. The leakage of LDH was measured in cell supernatants and viable cells were determined by MTT assay. Data are presented as mean + s.e.m. from 4 independent experiments. **(C)** HeLa cells were cultured for 24 h in serum-free medium with vehicle, 10 ng/ml 27-HC, 10 ng/ml 25-HC or 1 mM methyl-β-cyclodextrin (MβCD). Total cellular cholesterol was quantified and normalized to total protein. Data are presented as mean ± s.e.m. with dots representing the values from 4 independent experiments; statistical significance was determined by ANOVA with Dunnett’s *post hoc* test. **(D)** Representative Western blots of pyolysin binding and α-tubulin are shown for HeLa cells cultured for 24 h in serum-free medium with vehicle, 10 ng/ml 27-HC, or 50 nM T0901317 as a positive control. Densitometry data for pyolysin binding were normalized to the α-tubulin loading control and presented as mean + s.e.m. from 4 independent experiments; statistical significance was determined by ANOVA with Tukey’s *post hoc* test, ***P < 0.001, **P < 0.01. **(E)** HeLa cells were cultured for 16 h in serum-free medium with vehicle or 10 µM SZ58-035, washed twice with PBS, cultured for 24 h in control serum-free medium with vehicle, 10 ng/ml 27-HC or 10 ng/ml 25-HC, in combination with vehicle or 10 µM SZ58-035; and then challenged for 2 h with control serum-free medium (■) or 100 HU pyolysin (■). The leakage of LDH was measured in cell supernatants and viable cells were determined by MTT assay. Data are presented as mean + s.e.m. from 4 independent experiments; statistical significance was determined by ANOVA and Tukey’s *post hoc* test, ***P < 0.001, **P < 0.01, *P < 0.05. **(F)** HeLa cells were cultured for 24 h in serum-free medium with vehicle or 10 ng/ml 27-HC, treated for 30 min in serum-free medium with or without 100 mU/ml sphingomyelinase, and then challenged for 2 h with control serum-free medium (■) or 25 HU pyolysin (■). The leakage of LDH was measured in cell supernatants and viable cells were determined by MTT assay. Data are presented as mean + s.e.m. from 4 independent experiments; statistical significance was determined by ANOVA and Tukey’s *post hoc* test, ***P < 0.001.

We next explored other cholesterol regulation mechanisms that might explain hydroxycholesterol cytoprotection. We first examined whether culturing HeLa cells with additional cholesterol might diminish oxysterol cytoprotection, but the addition of 10% serum did not prevent treatment with 25-hydroxycholesterol protecting against a subsequent pyolysin challenge (**Figure 7A**). However, maximal cytoprotection required treatment with 100 ng/ml 25-hydroxycholesterol in the presence of serum, compared with 10 ng/ml in serum-free medium. As oxysterols have a similar structure to cholesterol, we also considered whether oxysterols might bind to pyolysin to prevent cytolysis. However, mixing pyolysin with 27-hydroxycholesterol or 25-hydroxycholesterol before challenging untreated cells did not diminish pyolysin-induced LDH-leakage or cytolysis (**Figure 7B**).

An additional mechanism that might be relevant is that 27-hydroxycholesterol stimulates inactivation of HMGCR, although the expectation would be that total cellular cholesterol abundance should also be reduced (34, 51). Inhibiting HMGCR with 10 µM atorvastatin reduced pyolysin-induced leakage of LDH and cytolysis (**Supplementary Figure 6A**) and reduced total cellular cholesterol (**Supplementary Figure 6B**). However, despite methyl-β-cyclodextrin reducing the abundance of HeLa cell cholesterol as expected, total cellular cholesterol was unaffected by 24 h treatment with 27-hydroxycholesterol (P = 0.97) or 25-hydroxycholesterol (P = 0.99, **Figure 7C**).

As side-chain hydroxycholesterols did not alter total cellular cholesterol, we investigated cell membrane accessible cholesterol. Treatment with 27-hydroxycholesterol reduced pyolysin binding to HeLa cells by 71%, as determined using an anti-pyolysin antibody (**Figure 7D**). Side-chain hydroxycholesterols stimulate ACAT esterification of cholesterol, which reduces accessible cholesterol in cell membranes (10, 28, 52, 53). Culturing HeLa cells with the ACAT inhibitor SZ58-035 for 16 h before and during 24 h treatment with 27-hydroxycholesterol or 25-hydroxycholesterol diminished cytoprotection against pyolysin-induced LDH leakage and cytolysis (**Figure 7E**). If cytoprotection depends on side-chain hydroxycholesterols reducing cell membrane accessible cholesterol, we reasoned that increasing this pool of cholesterol should also diminish cytoprotection. Accessible cholesterol can be generated using sphingomyelinase to release sphingomyelin-sequestered cholesterol (5, 10). Treating HeLa cells with sphingomyelinase both increased the sensitivity of cells to pyolysin damage, and diminished oxysterol cytoprotection against pyolysin (**Figure 7F**; P < 0.001). Together these data support the concept that side-chain hydroxycholesterols reduce accessible cholesterol in the cell membrane.

## 4 Discussion

We found that oxysterols stimulated the intrinsic protection of epithelial cells against damage caused by cholesterol-dependent cytolysins. Treatment with 27-hydroxycholesterol protected four types of epithelial cells against a subsequent challenge with pyolysin, and protected cells against streptolysin O and *Staphylococcus aureus* α-hemolysin. Treatment with 27-hydroxycholesterol reduced cytolysin-induced leakage of potassium ions and LDH, limited cytoskeletal changes, and reduced cytolysis. However, oxysterol cytoprotection did not appear to depend on potassium or calcium flux, or activation of LXRs. Instead, cytoprotection was dependent on ACAT reducing accessible cholesterol in cell membranes. Collectively, these findings imply that oxysterols stimulate the intrinsic protection of epithelial cells against pore-forming toxins and may help protect tissues against pathogenic bacteria. This oxysterol cytoprotection may help complement the role of oxysterols in modulating immune cell responses to bacteria (10, 27). We reason that protecting epithelial cells against pore-forming toxins is likely to be more evolutionary and energetically advantageous than repairing damage or mounting inflammatory responses. Preventing damage also helps tissues and organisms better tolerate the presence of pathogens (11). Finally, oxysterol cytoprotection might inspire new prophylactic applications that limit tissue damage caused by bacteria.

An important physiological role of the epithelium is to protect underlying tissue cells against bacterial infections (1, 11, 54). We found that pyolysin damaged several epithelial cell types, which is consistent with widespread effects of *T. pyogenes* and pyolysin across species and tissues (19–21, 24). However, our main finding was that treating HeLa cells with either 27-hydroxycholesterol or 25-hydroxycholesterol reduced pyolysin-induced cytolysis from > 68% to < 2%. There was also evidence of less pyolysin pore-formation with reduced pyolysin binding to cells, less potassium and LDH leakage, and prevention of pyolysin-induced MAPK phosphorylation. Side-chain hydroxycholesterol cytoprotection against pyolysin extended to A549, Hep-G2, and NCI-H441 epithelial cells. Although 25-hydroxycholesterol had no significant effect on *L. monocytogenes* escape from phagocytic vacuoles, which requires the cholesterol-dependent cytolysin listeriolysin O (10), we found 27-hydroxycholesterol also protected cells against streptolysin O. Our findings add to recent observations that interferon-stimulated production of 25-hydroxycholesterol protects murine macrophages against perfringolysin O, streptolysin O and anthrolysin O damage (28); and that 27-hydroxycholesterol or 25-hydroxycholesterol protects bovine endometrial epithelial and stromal cells against pyolysin (29). However, unlike the previous studies, we found that the ring-modified hydroxycholesterols 7α-hydroxycholesterol and 7β-hydroxycholesterol also partially protected HeLa cells against pyolysin, although they were not as potent as 27-hydroxycholesterol. Taking the evidence together, we suggest that oxysterols may contribute to maintaining or enhancing epithelial barriers against bacterial cytolysins. Further work is needed to determine the bioavailability of oxysterols in epithelial tissues and whether oxysterols regulate resilience to infection of epithelial barriers *in vivo*. However, oxysterols such as 27-hydroxycholesterol and 25-hydroxycholesterol are present in ng/ml amounts in the bovine endometrium, and 25-hydroxycholesterol is secreted by endometrial epithelial cells in response to lipopolysaccharide or pyolysin (29). Furthermore, injection of 25-hydroxycholesterol into the skin of mice reduced the tissue damaged caused by a subsequent injection of anthrolysin O (28).

Eukaryotic cells have evolved several mechanisms to respond to damage caused by pore-forming toxins (14, 16, 33). Cell membrane and cytoskeletal repair mechanisms are triggered by potassium efflux from cells and increased intracellular calcium (14–16). We therefore considered whether side-chain hydroxycholesterols may act by enhancing these ion fluxes to trigger damage repair responses, rather than protecting the cells against pore-formation. However, we did not find evidence that side-chain hydroxycholesterol cytoprotection was associated with changes in potassium or calcium. Thus, we suggest that hydroxycholesterols enhance the intrinsic protection of cells against damage, rather than improving their ability to recover from damage.

Reducing or modifying cell membrane cholesterol is an obvious mechanism for altering the intrinsic protection of cells against cholesterol-dependent cytolysins (3, 10, 21, 24, 26). There are three pools of cell membrane cholesterol: an essential pool, a sphingomyelin-sequestered pool, and a labile pool of accessible cholesterol that can be bound by cholesterol-dependent cytolysins (5, 25). Activation of LXRs can stimulate cholesterol efflux from cells (49), and the LXR agonist T0901317 partially protected HeLa cells against pyolysin in the present study. Similarly, another synthetic LXR agonist, GW3965, also protects macrophages against cholesterol-dependent cytolysin damage (28). Although, side-chain hydroxycholesterols are LXR agonists (49, 55), using siRNA to target *NR1H2* and *NR1H3* did not diminish oxysterol cytoprotection in the present study. At micromolar concentrations, 27-hydroxycholesterol can also act *via* estrogen receptors, but this mechanism is unlikely to be important for the present study because estrogen receptors are not expressed by HeLa or A549 cells (56, 57). We also considered whether 27-hydroxycholesterol inactivation of HMGCR might be important because inhibiting HMGCR protects bovine endometrial stromal cells against pyolysin (34). Surprisingly, 27-hydroxycholesterol and 25-hydroxycholesterol did not reduce total cellular cholesterol, as expected when inhibiting HMGCR (34, 51). However, this observation concurs with a similar finding using Chinese hamster ovary cells treated with 2 µg/ml 25-hydroxycholesterol for 20 h (10). Instead, we found that 27-hydroxycholesterol reduced pyolysin binding to cells, which is consistent with less accessible cholesterol in the cell membrane (4). Side-chain hydroxycholesterols reduce accessible cholesterol in cell membranes by stimulating ACAT esterification of cholesterol (10, 28, 53). In agreement with this concept, oxysterol cytoprotection against pyolysin was diminished in the present study by inhibiting cholesterol esterification or by releasing sphingomyelin-bound cholesterol. However, as some cytoprotection against pyolysin remained in these experiments, oxysterol-stimulated ACAT cholesterol esterification may not be the sole mechanism for cytoprotection. For example, we cannot rule out the possibility that oxysterols may protect against cytolysins by inhibiting cholesterol biosynthesis as a result of oxysterol binding to INSIG to prevent processing of SREB2 (26).

It was intriguing that treatment with 27-hydroxycholesterol also protected HeLa and A549 cells against *Staphylococcus aureus* α-hemolysin. A longer challenge period was required to induce α-hemolysin cytolysis compared with pyolysin, possibly due to the ten-fold smaller pore-diameter (22, 46). Conversely, these smaller pores have a lower calcium permeability than cholesterol-dependent cytolysins, which can impair calcium-dependent protective responses (58, 59). A further benefit of oxysterols for limiting the severity of disease is that oxysterols can also suppresses macrophage inflammatory response to pathogens (8, 27). Therefore, future *in vivo* studies might test the prophylactic effect of oxysterol treatment on the severity of *Staphylococcus aureus* infection, as well as infections caused by bacteria producing cholesterol-dependent cytolysins.

In conclusion, we found that oxysterols stimulated the intrinsic protection of epithelial cells against damage caused by cholesterol-dependent cytolysins. In particular, treatment with 27-hydroxycholesterol protected HeLa cells against a subsequent challenge with pyolysin or streptolysin O. Oxysterol cytoprotection extended to other cell types and protected against *Staphylococcus aureus* α-hemolysin. Mechanistically, oxysterol cytoprotection was at least partially dependent on ACAT reducing accessible cholesterol in the cell membrane. Collectively, these findings imply that oxysterols stimulate the intrinsic protection of epithelial cells against pore-forming toxins and may help protect tissues against pathogenic bacteria.

## Data Availability Statement

The original contributions presented in the study are included in the article/**Supplementary Material**. Further inquiries can be directed to the corresponding author.

## Author Contributions

Conceptualization, IS, SO, and TO. Methodology, IS, SO, and TO. Investigation TO, LC, TM, and SO. Writing - original draft preparation TO and IS. Writing - review and editing IS, TO, SO, LC, TM, JC, and JB. Visualization TO, IS, and SO. Supervision, IS, JC, and JB. Project administration IS. Funding acquisition IS and JB. All authors contributed to the article and approved the submitted version.

## Funding

This study was supported in part by the Eunice Kennedy Shriver National Institute of Child Health & Human Development of the National Institutes of Health under Award Number R01HD084316. The content is solely the responsibility of the authors and does not necessarily represent the official views of the National Institutes of Health.

## Conflict of Interest

The authors declare that the research was conducted in the absence of any commercial or financial relationships that could be construed as a potential conflict of interest.

## Publisher’s Note

All claims expressed in this article are solely those of the authors and do not necessarily represent those of their affiliated organizations, or those of the publisher, the editors and the reviewers. Any product that may be evaluated in this article, or claim that may be made by its manufacturer, is not guaranteed or endorsed by the publisher.

## Abbreviations

27-HC, 27-hydroxycholesterol ((25R)26-hydroxycholesterol); 25-HC, 25-hydroxycholesterol; ACAT, acyl-CoA:cholesterol acyltransferase; HMGCR, 3-hydroxy-3-methyl-glutaryl-coenzyme A reductase; HU, hemolytic units; LDH, lactate dehydrogenase; LXR, Liver X receptor; MAPK, mitogen-activated protein kinase.

## References

[1] Los FC Randis TM Aroian RV Ratner AJ. Role of Pore-Forming Toxins in Bacterial Infectious Diseases. Microbiol Mol Biol Rev (2013) 77(2):173–207. doi: 10.1128/MMBR.00052-12 23699254 PMC3668673

[2] Dal Peraro M van der Goot FG. Pore-Forming Toxins: Ancient, But Never Really Out of Fashion. Nat Rev: Microbiol (2016) 14(2):77–92. doi: 10.1038/nrmicro.2015.3 26639780

[3] Tweten RK. Cholesterol-Dependent Cytolysins, a Family of Versatile Pore-Forming Toxins. Infect Immun (2005) 73(10):6199–209. doi: 10.1128/IAI.73.10.6199-6209.2005 PMC1230961 16177291

[4] Das A Goldstein JL Anderson DD Brown MS Radhakrishnan A. Use of Mutant 125I-Perfringolysin O to Probe Transport and Organization of Cholesterol in Membranes of Animal Cells. Proc Natl Acad Sci USA (2013) 110(26):10580–5. doi: 10.1073/pnas.1309273110 PMC3696761 23754385

[5] Das A Brown MS Anderson DD Goldstein JL Radhakrishnan A. Three Pools of Plasma Membrane Cholesterol and Their Relation to Cholesterol Homeostasis. eLife (2014) 3:e02882, 02881–02816. doi: 10.7554/eLife.02882 PMC4086274 24920391

[6] Bielska AA Olsen BN Gale SE Mydock-McGrane L Krishnan K Baker NA. Side-Chain Oxysterols Modulate Cholesterol Accessibility Through Membrane Remodeling. Biochemistry (2014) 53(18):3042–51. doi: 10.1021/bi5000096 PMC4020583 24758724

[7] Brown AJ Sharpe LJ Rogers MJ. Oxysterols: From Physiological Tuners to Pharmacological Opportunities. Br J Pharmacol (2021) 178:3089–103. doi: 10.1111/bph.15073 32335907

[8] Reboldi A Dang EV McDonald JG Liang G Russell DW Cyster JG. 25-Hydroxycholesterol Suppresses Interleukin-1-Driven Inflammation Downstream of Type I Interferon. Science (2014) 345(6197):679–84. doi: 10.1126/science.1254790 PMC4289637 25104388

[9] Dang EV McDonald JG Russell DW Cyster JG. Oxysterol Restraint of Cholesterol Synthesis Prevents AIM2 Inflammasome Activation. Cell (2017) 171(5):1057–71. doi: 10.1016/j.cell.2017.09.029 PMC5693620 29033131

[10] Abrams ME Johnson KA Perelman SS Zhang LS Endapally S Mar KB. Oxysterols Provide Innate Immunity to Bacterial Infection by Mobilizing Cell Surface Accessible Cholesterol. Nat Microbiol (2020) 5(7):929–42. doi: 10.1038/s41564-020-0701-5 PMC7442315 32284563

[11] Medzhitov R Schneider DS Soares MP. Disease Tolerance as a Defense Strategy. Science (2012) 335(6071):936–41. doi: 10.1126/science.1214935 PMC3564547 22363001

[12] Huffman DL Abrami L Sasik R Corbeil J van der Goot FG Aroian RV. Mitogen-Activated Protein Kinase Pathways Defend Against Bacterial Pore-Forming Toxins. Proc Natl Acad Sci USA (2004) 101(30):10995–1000. doi: 10.1073/pnas.0404073101 PMC503732 15256590

[13] Babiychuk EB Monastyrskaya K Potez S Draeger A. Intracellular Ca(2+) Operates a Switch Between Repair and Lysis of Streptolysin O-Perforated Cells. Cell Death Differ (2009) 16(8):1126–34. doi: 10.1038/cdd.2009.30 19325569

[14] Gonzalez MR Bischofberger M Freche B Ho S Parton RG van der Goot FG. Pore-Forming Toxins Induce Multiple Cellular Responses Promoting Survival. Cell Microbiol (2011) 13(7):1026–43. doi: 10.1111/j.1462-5822.2011.01600.x 21518219

[15] Wippel C Fortsch C Hupp S Maier E Benz R Ma J. Extracellular Calcium Reduction Strongly Increases the Lytic Capacity of Pneumolysin From Streptococcus Pneumoniae in Brain Tissue. J Infect Dis (2011) 204(6):930–6. doi: 10.1093/infdis/jir434 PMC3156923 21849290

[16] Andrews NW Corrotte M. Plasma Membrane Repair. Curr Biol (2018) 28(8):R392–7. doi: 10.1016/j.cub.2017.12.034 29689221

[17] Turner ML Owens SE Sheldon IM. Glutamine Supports the Protection of Tissue Cells Against the Damage Caused by Cholesterol-Dependent Cytolysins From Pathogenic Bacteria. PloS One (2020) 15(3):e0219275. doi: 10.1371/journal.pone.0219275 32163417 PMC7067430

[18] Pospiech M Owens SE Miller DJ Austin-Muttitt K Mullins JGL Cronin JG. Bisphosphonate Inhibitors of Squalene Synthase Protect Cells Against Cholesterol-Dependent Cytolysins. FASEB J (2021) 35(6):e21640. doi: 10.1096/fj.202100164R 33991130 PMC12315948

[19] Billington SJ Jost BH Cuevas WA Bright KR Songer JG. The Arcanobacterium (Actinomyces) Pyogenes Hemolysin, Pyolysin, is a Novel Member of the Thiol-Activated Cytolysin Family. J Bacteriol (1997) 179(19):6100–6. doi: 10.1128/jb.179.19.6100-6106.1997 PMC179514 9324258

[20] Jost BH Billington SJ. Arcanobacterium Pyogenes: Molecular Pathogenesis of an Animal Opportunist. Antonie Van Leeuwenhoek (2005) 88(2):87–102. doi: 10.1007/s10482-005-2316-5 16096685

[21] Amos MR Healey GD Goldstone RJ Mahan S Duvel A Schuberth HJ. Differential Endometrial Cell Sensitivity to a Cholesterol-Dependent Cytolysin Links Trueperella Pyogenes to Uterine Disease in Cattle. Biol Reprod (2014) 90(3):54. doi: 10.1095/biolreprod.113.115972 24478394

[22] Preta G Jankunec M Heinrich F Griffin S Sheldon IM Valincius G. Tethered Bilayer Membranes as a Complementary Tool for Functional and Structural Studies: The Pyolysin Case. Biochim Biophys Acta (BBA) - Biomembr (2016) 1858(9):2070–80. doi: 10.1016/j.bbamem.2016.05.016 27211243

[23] Giddings KS Johnson AE Tweten RK. Redefining Cholesterol’s Role in the Mechanism of the Cholesterol-Dependent Cytolysins. Proc Natl Acad Sci USA (2003) 100(20):11315–20. doi: 10.1073/pnas.2033520100 PMC208754 14500900

[24] Preta G Lotti V Cronin JG Sheldon IM. Protective Role of the Dynamin Inhibitor Dynasore Against the Cholesterol-Dependent Cytolysin of Trueperella Pyogenes. FASEB J (2015) 29(4):1516–28. doi: 10.1096/fj.14-265207 PMC4396600 25550455

[25] Endapally S Frias D Grzemska M Gay A Tomchick DR Radhakrishnan A. Molecular Discrimination Between Two Conformations of Sphingomyelin in Plasma Membranes. Cell (2019) 176(5):1040–53. doi: 10.1016/j.cell.2018.12.042 PMC6428426 30712872

[26] Griffiths WJ Wang Y. Sterols, Oxysterols, and Accessible Cholesterol: Signalling for Homeostasis, in Immunity and During Development. Front Physiol (2021) 12:723224. doi: 10.3389/fphys.2021.723224 34690800 PMC8531217

[27] Cyster JG Dang EV Reboldi A Yi T. 25-Hydroxycholesterols in Innate and Adaptive Immunity. Nat Rev: Immunol (2014) 14(11):731–43. doi: 10.1038/nri3755 25324126

[28] Zhou QD Chi X Lee MS Hsieh WY Mkrtchyan JJ Feng A-C. Interferon-Mediated Reprogramming of Membrane Cholesterol to Evade Bacterial Toxins. Nat Immunol (2020) 21(7):746–55. doi: 10.1038/s41590-020-0695-4 PMC7778040 32514064

[29] Ormsby TJR Owens SE Horlock AD Davies D Griffiths WJ Wang Y. Oxysterols Protect Bovine Endometrial Cells Against Pore-Forming Toxins From Pathogenic Bacteria. FASEB J (2021) 35(10):e21889. doi: 10.1096/fj.202100036R 34569656 PMC9272411

[30] Griffin S Healey GD Sheldon IM. Isoprenoids Increase Bovine Endometrial Stromal Cell Tolerance to the Cholesterol-Dependent Cytolysin From Trueperella Pyogenes. Biol Reprod (2018) 99(4):749–60. doi: 10.1093/biolre/ioy099 PMC6203874 29688258

[31] Mestre MB Colombo MI. cAMP and EPAC are Key Players in the Regulation of the Signal Transduction Pathway Involved in the Alpha-Hemolysin Autophagic Response. PloS Pathog (2012) 8(5):e1002664. doi: 10.1371/journal.ppat.1002664 22654658 PMC3359991

[32] Schultz JR Tu H Luk A Repa JJ Medina JC Li L. Role of LXRs in Control of Lipogenesis. Genes Dev (2000) 14(22):2831–8. doi: 10.1101/gad.850400 PMC317060 11090131

[33] Gurcel L Abrami L Girardin S Tschopp J van der Goot FG. Caspase-1 Activation of Lipid Metabolic Pathways in Response to Bacterial Pore-Forming Toxins Promotes Cell Survival. Cell (2006) 126(6):1135–45. doi: 10.1016/j.cell.2006.07.033 16990137

[34] Griffin S Preta G Sheldon IM. Inhibiting Mevalonate Pathway Enzymes Increases Stromal Cell Resilience to a Cholesterol-Dependent Cytolysin. Sci Rep (2017) 7(1):17050. doi: 10.1038/s41598-017-17138-y 29213055 PMC5719056

[35] Ross AC Go KJ Heider JG Rothblat GH. Selective Inhibition of Acyl Coenzyme A:cholesterol Acyltransferase by Compound 58-035. J Biol Chem (1984) 259(2):815–9. doi: 10.1016/S0021-9258(17)43530-7 6693397

[36] Bustin SA Benes V Garson JA Hellemans J Huggett J Kubista M. The MIQE Guidelines: Minimum Information for Publication of Quantitative Real-Time PCR Experiments. Clin Chem (2009) 55(4):611–22. doi: 10.1373/clinchem.2008.112797 19246619

[37] Bromfield JJ Sheldon IM. Lipopolysaccharide Initiates Inflammation in Bovine Granulosa Cells via the TLR4 Pathway and Perturbs Oocyte Meiotic Progression In Vitro. Endocrinology (2011) 152(12):5029–40. doi: 10.1210/en.2011-1124 PMC3428914 21990308

[38] Schindelin J Arganda-Carreras I Frise E Kaynig V Longair M Pietzsch T. Fiji: An Open-Source Platform for Biological-Image Analysis. Nat Methods (2012) 9(7):676–82. doi: 10.1038/nmeth.2019 PMC3855844 22743772

[39] Brown MS Goldstein JL. A Receptor-Mediated Pathway for Cholesterol Homeostasis. Science (1986) 232(4746):34–47. doi: 10.1126/science.3513311 3513311

[40] Renshaw MW Ren XD Schwartz MA. Growth Factor Activation of MAP Kinase Requires Cell Adhesion. EMBO J (1997) 16(18):5592–9. doi: 10.1093/emboj/16.18.5592 PMC1170191 9312018

[41] Dzeletovic S Breuer O Lund E Diczfalusy U. Determination of Cholesterol Oxidation Products in Human Plasma by Isotope Dilution-Mass Spectrometry. Anal Biochem (1995) 225(1):73–80. doi: 10.1006/abio.1995.1110 7778789

[42] Ratner AJ Hippe KR Aguilar JL Bender MH Nelson AL. Epithelial Cells Are Sensitive Detectors of Bacterial Pore-Forming Toxins. J Biol Chem (2006) 281(18):12994–8. doi: 10.1074/jbc.M511431200 PMC1586115 16520379

[43] Statt S Ruan JW Hung LY Chang CY Huang CT Lim JH. Statin-Conferred Enhanced Cellular Resistance Against Bacterial Pore-Forming Toxins in Airway Epithelial Cells. Am J Respir Cell Mol Biol (2015) 53(5):689–702. doi: 10.1165/rcmb.2014-0391OC 25874372 PMC4742951

[44] Bhakdi S Tranumjensen J Sziegoleit A. Mechanism of Membrane Damage by Streptolysin-O. Infect Immun (1985) 47(1):52–60. doi: 10.1128/iai.47.1.52-60.1985 3880730 PMC261464

[45] Keyel PA Loultcheva L Roth R Salter RD Watkins SC Yokoyama WM. Streptolysin O Clearance Through Sequestration Into Blebs That Bud Passively From the Plasma Membrane. J Cell Sci (2011) 124(Pt 14):2414–23. doi: 10.1242/jcs.076182 PMC3124372 21693578

[46] Song L Hobaugh MR Shustak C Cheley S Bayley H Gouaux JE. Structure of Staphylococcal Alpha-Hemolysin, a Heptameric Transmembrane Pore. Science (1996) 274(5294):1859–66. doi: 10.1126/science.274.5294.1859 8943190

[47] Seilie ES Bubeck Wardenburg J. Staphylococcus Aureus Pore-Forming Toxins: The Interface of Pathogen and Host Complexity. Semin Cell Dev Biol (2017) 72:101–16. doi: 10.1016/j.semcdb.2017.04.003 PMC5823538 28445785

[48] Lemaire-Ewing S Berthier A Royer MC Logette E Corcos L Bouchot A. 7beta-Hydroxycholesterol and 25-Hydroxycholesterol-Induced Interleukin-8 Secretion Involves a Calcium-Dependent Activation of C-Fos via the ERK1/2 Signaling Pathway in THP-1 Cells: Oxysterols-Induced IL-8 Secretion is Calcium-Dependent. Cell Biol Toxicol (2009) 25(2):127–39. doi: 10.1007/s10565-008-9063-0 18317936

[49] Wang B Tontonoz P. Liver X Receptors in Lipid Signalling and Membrane Homeostasis. Nat Rev: Endocrinol (2018) 14(8):452–63. doi: 10.1038/s41574-018-0037-x PMC6433546 29904174

[50] Fu X Menke JG Chen Y Zhou G MacNaul KL Wright SD. 27-Hydroxycholesterol is an Endogenous Ligand for Liver X Receptor in Cholesterol-Loaded Cells. J Biol Chem (2001) 276(42):38378–87. doi: 10.1074/jbc.M105805200 11504730

[51] Lange Y Ory DS Ye J Lanier MH Hsu F-F Steck TL. Effectors of Rapid Homeostatic Responses of Endoplasmic Reticulum Cholesterol and 3-Hydroxy-3-Methylglutaryl-CoA Reductase. J Biol Chem (2008) 283(3):1445–55. doi: 10.1074/jbc.M706967200 18024962

[52] Chang TY Li BL Chang CC Urano Y. Acyl-Coenzyme A:cholesterol Acyltransferases. Am J Physiol: Endocrinol Metab (2009) 297(1):E1–9. doi: 10.1152/ajpendo.90926.2008 PMC2711667 19141679

[53] Wang S Li W Hui H Tiwari SK Zhang Q Croker BA. Cholesterol 25-Hydroxylase Inhibits SARS-CoV-2 and Other Coronaviruses by Depleting Membrane Cholesterol. EMBO J (2020) 39(21):e106057. doi: 10.15252/embj.2020106057 32944968 PMC7537045

[54] Bischofberger M Gonzalez MR van der Goot FG. Membrane Injury by Pore-Forming Proteins. Curr Opin Cell Biol (2009) 21(4):589–95. doi: 10.1016/j.ceb.2009.04.003 19442503

[55] Lehmann JM Kliewer SA Moore LB Smith-Oliver TA Oliver BB Su JL. Activation of the Nuclear Receptor LXR by Oxysterols Defines a New Hormone Response Pathway. J Biol Chem (1997) 272(6):3137–40. doi: 10.1074/jbc.272.6.3137 9013544

[56] DuSell CD Umetani M Shaul PW Mangelsdorf DJ McDonnell DP. 27-Hydroxycholesterol is an Endogenous Selective Estrogen Receptor Modulator. Mol Endocrinol (2008) 22(1):65–77. doi: 10.1210/me.2007-0383 17872378 PMC2194632

[57] Niikawa H Suzuki T Miki Y Suzuki S Nagasaki S Akahira J. Intratumoral Estrogens and Estrogen Receptors in Human Non-Small Cell Lung Carcinoma. Clin Cancer Res (2008) 14(14):4417–26. doi: 10.1158/1078-0432.Ccr-07-1950 18579664

[58] Walev I Martin E Jonas D Mohamadzadeh M Muller-Klieser W Kunz L. Staphylococcal Alpha-Toxin Kills Human Keratinocytes by Permeabilizing the Plasma Membrane for Monovalent Ions. Infect Immun (1993) 61(12):4972–9. doi: 10.1128/IAI.61.12.4972-4979.1993 PMC281271 8225571

[59] Brito C Cabanes D Sarmento Mesquita F Sousa S. Mechanisms Protecting Host Cells Against Bacterial Pore-Forming Toxins. Cell Mol Life Sci (2019) 76(7):1319–39. doi: 10.1007/s00018-018-2992-8 PMC6420883 30591958

