# Supplementary material for: Oxysterols Protect Epithelial Cells Against Pore-Forming Toxins
Source: Front Immunol. 2022 Jan 26;13:815775. doi: 10.3389/fimmu.2022.815775 (PMC8825411; doi:10.3389/fimmu.2022.815775)
Supplement: Supplementary file 1 [file DataSheet_1.pdf]

Supplementary Information

Supplementary Table 1. siRNA sequences for target genes

Gene	Direction	Sequence (5'–3')
NR1H2	Sense	GAAGAAGAAGAUUCGGAAAUU
	Antisense	UUUCCGAAUCUUCUUCUUC
NR1H3	Sense	CCUCAAGGAUUUCAGUUAUUU
	Antisense	AUAACUGAAAUCCUUGAGG

Supplementary Table 2. Primer Sequences

**Gene**	**Direction**	**Sequence (5'–3')**
RPL19	Forward	GCGAGCTCTTTCCTTTCGCT
	Reverse	TGCTGACGGGAGTTGGCATT
NR1H2	Forward	CAGACTGGGGCGTCCTTTC
	Reverse	GACTGCGACTGTGACTGTGA
NR1H3	Forward	GGAGGTACAACCCTGGGAGT
	Reverse	AGCAATGAGCAAGGCAAACT

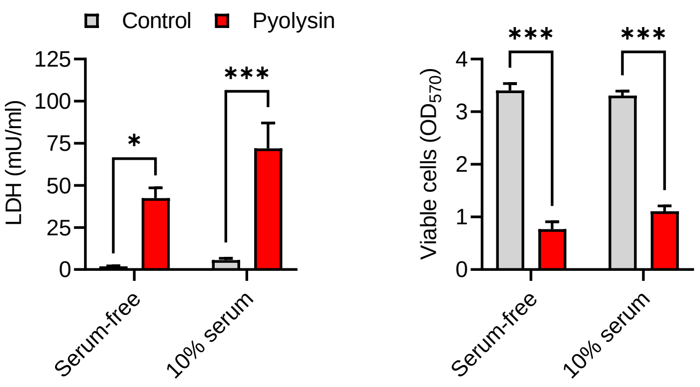

Supplementary Figure 1. Serum-free culture does not prevent pyolysin damage to epithelial cells

HeLa cervical cells were cultured for 24 h in serum-free medium or medium containing 10% fetal bovine serum, and then challenged for 2 h in serum-free medium with 100 HU pyolysin. The leakage of LDH was measured in cell supernatants and viable cells were determined by MTT assay. Data are presented as mean + s.e.m. from 6 independent experiments; statistical significance was determined using two-way ANOVA and Šídák's multiple comparisons test, *** $P < 0.001$, * $P <0.05$

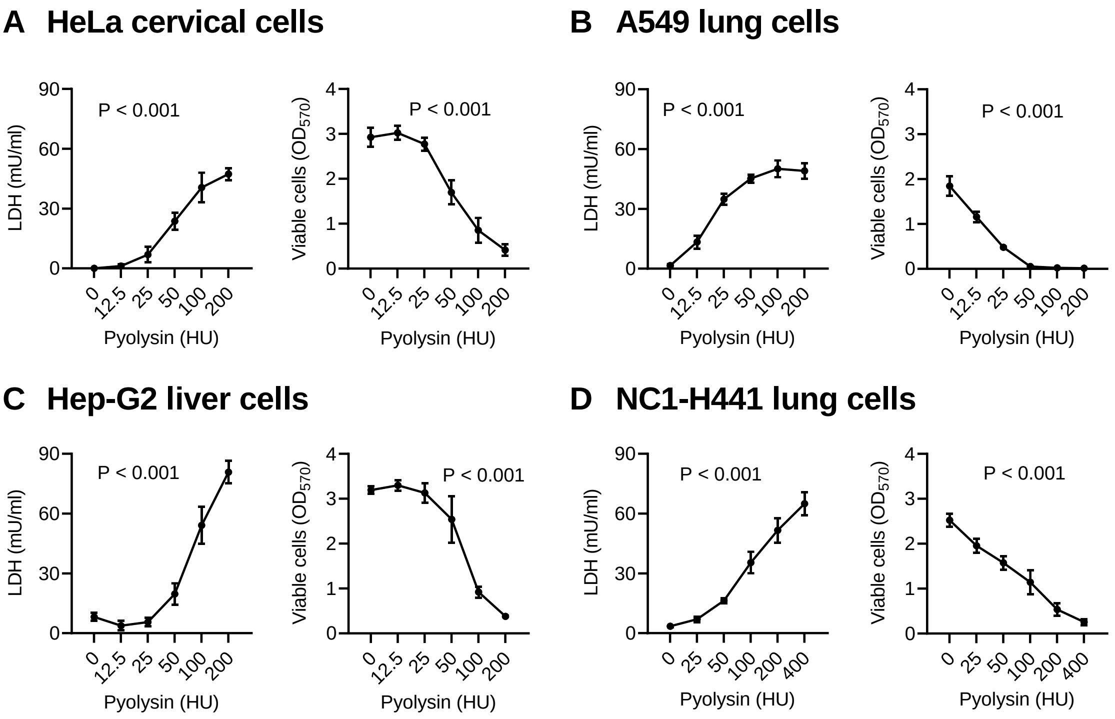

Supplementary Figure 2. Pyolysin damages epithelial cells

(A) HeLa cervical cells, (B) A549 lung cells, (C) Hep-G2 liver cells or (D) NC1-H441 normal lung cells were cultured for 24 h in serum-free medium and then challenged for 2 h in serum-free medium with the indicated amounts of pyolysin (HU, hemolytic units). The leakage of LDH was measured in cell supernatants and viable cells were determined by MTT assay. Data are presented as mean ± s.e.m. from 4 independent experiments; statistical significance was determined using ANOVA and P-values reported.

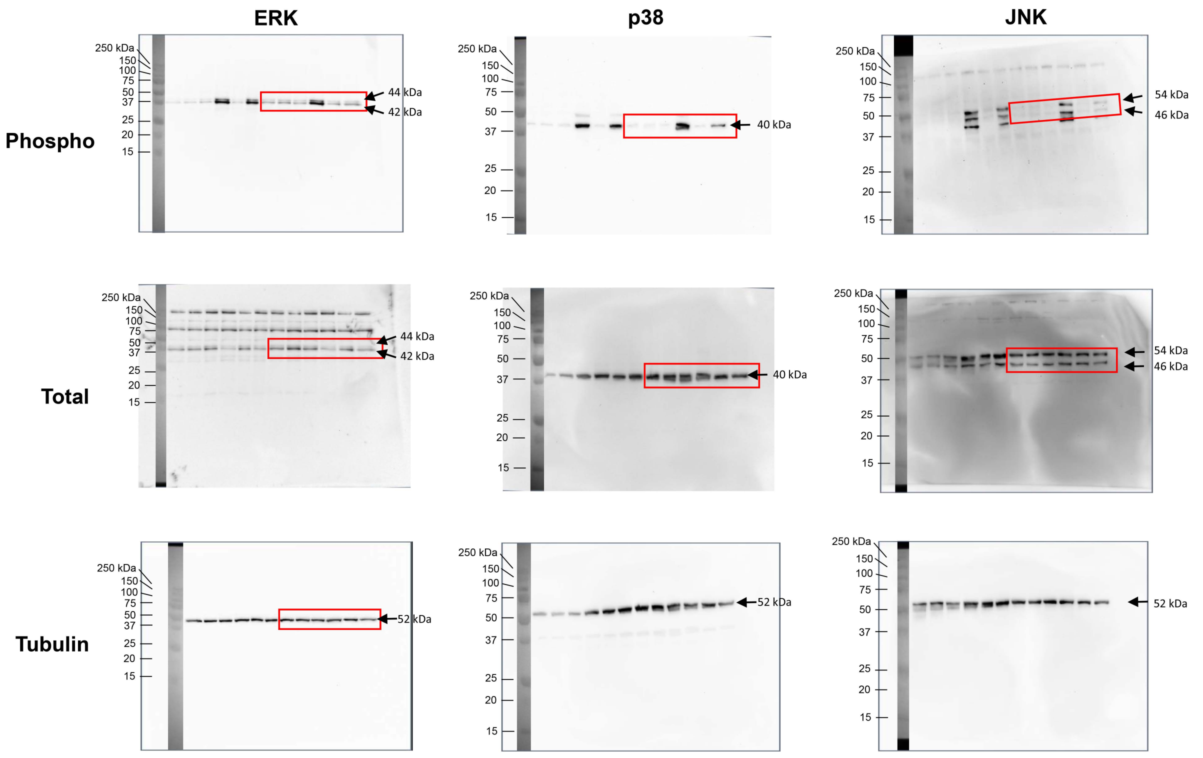

Supplementary Figure 3. MAPK phosphorylation

Representative whole Western blots used in Figure 3 (red boxes), displaying phosphorylated and total ERK1/2, p38 and JNK, and α-tubulin for HeLa cells treated with vehicle, 10 ng/ml 27-hydroxycholesterol (27-HC) or 50 nM T0901317 for 24 h, and challenged with control serum-free medium or 100 HU pyolysin for 10 min. Precision Plus All Blue Protein Standards (Bio-Rad) were loaded in the first lane for each whole blot, and sample proteins loaded in the remaining lanes. We then capture a template image with the membrane illuminated first with the epi-white light to display the protein standards (ChemiDoc XRS System, Bio-Rad). Without moving the membrane, a second chemiluminescence image is captured displaying the target immunoblot bands of the samples. Therefore, the protein standards image and chemiluminescence image are both captured from the same whole blot in the same position. The figure shows each pair of images overlaid, but with the lane containing the protein standards in the chemiluminescence image cropped out, so that the underlying protein standards image is visible.

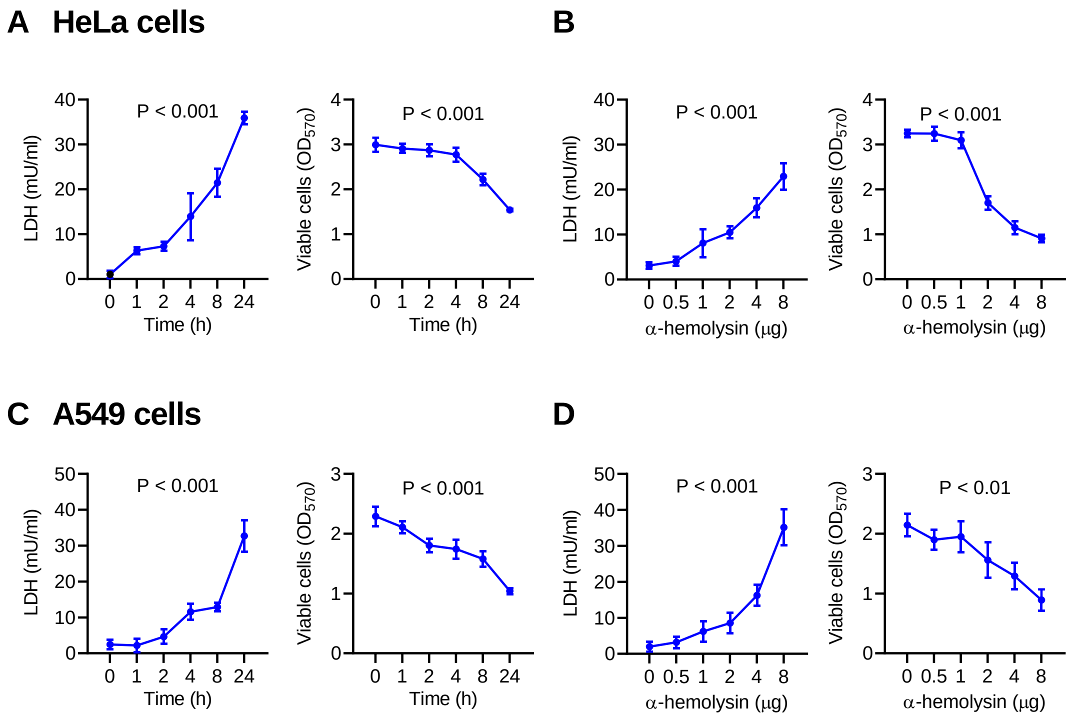

Supplementary Figure 4. ***Staphylococcus aureus*** **α-hemolysin damages epithelial cells**

(A, B) HeLa and (C, D) A549 cells were cultured for 24 h in serum-free medium and then challenged in serum-free medium with (A, C) 8 μg/well α-hemolysin for the indicated times, or (B, D) the indicated amounts of α-hemolysin for 24 h. The leakage of LDH was measured in cell supernatants and viable cells were determined by MTT assay. Data are presented as mean ± s.e.m. from 4 independent experiments; statistical significance was determined using ANOVA and P-values reported.

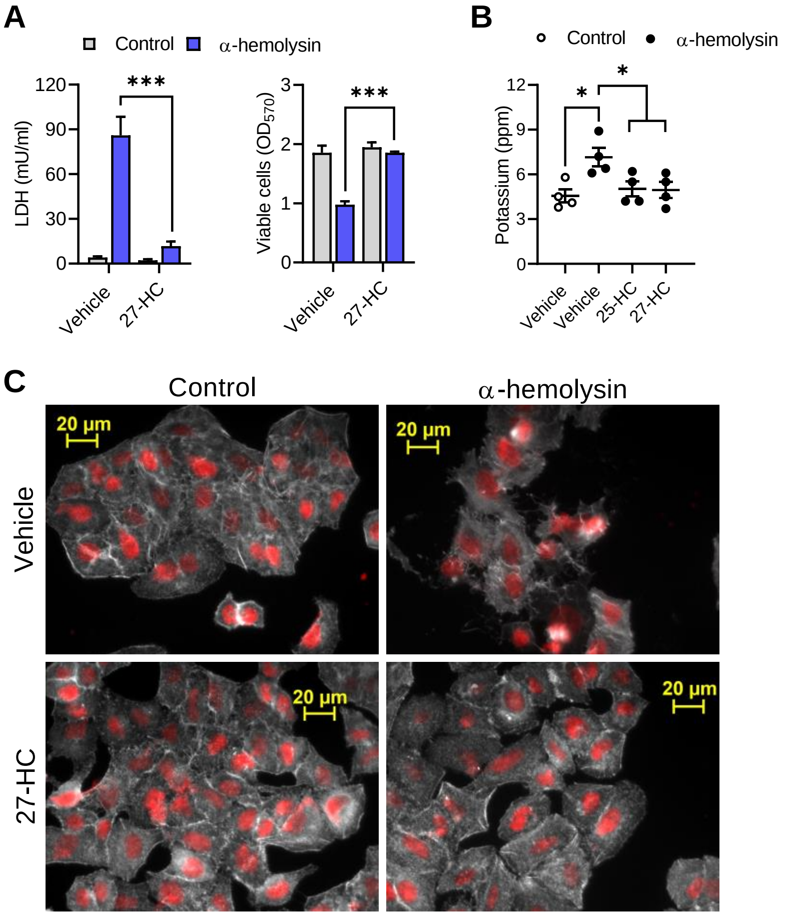

Supplementary Figure 5. Oxysterols protect A549 cells against *Staphylococcus aureus* α-hemolysin

(A) A549 cells were cultured for 24 h in serum-free medium with vehicle or 25 ng/ml 27-hydroxycholesterol (27-HC), and then challenged for 24 h with control medium (■) or 8 µg/well α-hemolysin (■). The leakage of LDH was measured in cell supernatants and viable cells were determined by MTT assay. Data are presented as mean + s.e.m. from 4 independent experiments; statistical significance was determined by ANOVA and Tukey's post hoc test, *** P < 0.001. (B) A549 cells were cultured for 24 h in serum-free medium with vehicle, 25 ng/ml 27-HC or 50 ng/ml 25-hydroxycholesterol (25-HC), and then challenged for 15 min with control serum-free medium (○) or 8 µg/well α-hemolysin (●). Extracellular potassium was measured in supernatants by flame photometry. Data are presented as mean ± s.e.m. with dots representing the values from 4 independent experiments; statistical significance was determined by ANOVA and Dunnett's post hoc test. (C) Fluorescent microscope images of A549 cells cultured for 24 h in serum-free medium with vehicle or 25 ng/ml 27-HC, and then challenged for 2 h with control medium or 8 µg/well α-hemolysin. Cells were stained with Alexa Fluor 555-conjugated phalloidin to visualize F-actin (white) and fluorescent microscope images collected (nuclei are red); images are representative of 3 experiments.

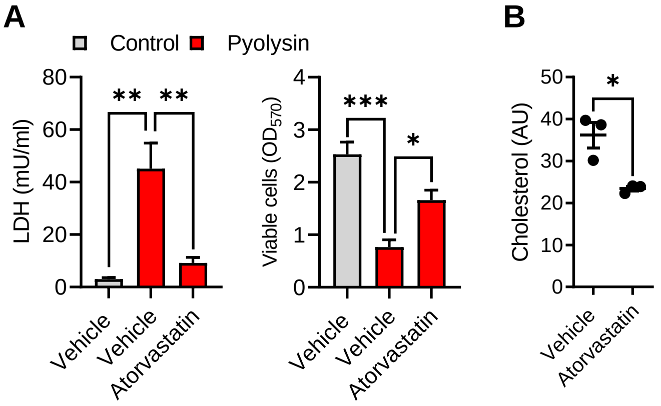

Supplementary Figure 6. Cellular cholesterol and cytoprotection

(A) HeLa cells were cultured for 24 h in serum-free medium with vehicle or 10 µM atorvastatin, and then challenged for 2 h with control serum-free medium (■) or 100 HU pyolysin (■). The leakage of LDH was measured in cell supernatants and viable cells were determined by MTT assay. Data are presented as mean + s.e.m. from 4 independent experiments; statistical significance was determined by ANOVA with Dunnett's post hoc test, *** $P < 0.001$, ** $P < 0.01$, * $P < 0.05$. (B) HeLa cells were cultured for 24 h in serum-free medium with vehicle or 10 µM atorvastatin. Total cellular cholesterol was quantified and normalized to total protein concentration. Data are presented as mean ± s.e.m. with dots representing independent experiments; statistical significance was determined by independent t-test, * $P < 0.05$
